# Clustering reveals limits of parameter identifiability in multi-parameter models of biochemical dynamics

**DOI:** 10.1186/s12918-015-0205-8

**Published:** 2015-09-29

**Authors:** Karol Nienałtowski, Michał Włodarczyk, Tomasz Lipniacki, Michał Komorowski

**Affiliations:** Institute of Fundamental Technological Research, Polish Academy of Sciences, Warsaw, Poland; Faculty of Mathematics Informatics and Mechanics, University of Warsaw, Warsaw, Poland

## Abstract

**Background:**

Compared to engineering or physics problems, dynamical models in quantitative biology typically depend on a relatively large number of parameters. Progress in developing mathematics to manipulate such multi-parameter models and so enable their efficient interplay with experiments has been slow. Existing solutions are significantly limited by model size.

**Results:**

In order to simplify analysis of multi-parameter models a method for clustering of model parameters is proposed. It is based on a derived statistically meaningful measure of similarity between groups of parameters. The measure quantifies to what extend changes in values of some parameters can be compensated by changes in values of other parameters. The proposed methodology provides a natural mathematical language to precisely communicate and visualise effects resulting from compensatory changes in values of parameters. As a results, a relevant insight into identifiability analysis and experimental planning can be obtained. Analysis of NF- *κ*B and MAPK pathway models shows that highly compensative parameters constitute clusters consistent with the network topology. The method applied to examine an exceptionally rich set of published experiments on the NF- *κ*B dynamics reveals that the experiments jointly ensure identifiability of only 60 % of model parameters. The method indicates which further experiments should be performed in order to increase the number of identifiable parameters.

**Conclusions:**

We currently lack methods that simplify broadly understood analysis of multi-parameter models. The introduced tools depict mutually compensative effects between parameters to provide insight regarding role of individual parameters, identifiability and experimental design. The method can also find applications in related methodological areas of model simplification and parameters estimation.

**Electronic supplementary material:**

The online version of this article (doi:10.1186/s12918-015-0205-8) contains supplementary material, which is available to authorized users.

## Background

Methods to understand the relationship between parameters (input) and model properties (output) are of particular interest in the context of biochemical dynamics and related phenomena. Sensitivity analysis and statistical inference have proven their importance for utilising modelling in physics and engineering. Models of biochemical dynamics, however, are different from conventional models in a number of ways. Primarily they involve a substantially larger number of parameters compared to available data. The high number of parameters and sparse data in ordinary differential equation (ODE) models make a conventional sensitivity analysis and statistical inference methods often prohibitively difficult to apply. This challenge has given rise to a number of approaches aimed at improving our ability to develop, verify and manipulate multi-parameter mechanistic models of such systems. These methods can be vaguely grouped into those aiming at: 1) improved description of parameter sensitivities; 2) detection of parameters that cannot be inferred from experimental data (identifiability analysis) and 3) guided experimental design to improve parameter identifiability and inference accuracy. Within the first group a number of studies have reported an intrinsic feature of dynamic multi-parameter models of biochemical dynamics to be sensitive only to a small number of linear combinations of parameters [1–5]. The conventional identifiability analysis verifies whether local distinct changes in parameter values imply distinct changes in model behaviour. A priori methods focus on determining whether this condition is satisfied prior to data collection. This can be done either based on model structure, often by attempting to find functional relationships between parameters [6], or by analysing model responses to local perturbations in parameter values. The latter is achieved by examining the Fisher information matrix (FIM). Two natural sources of non-identifiability have been recognised: insensitivity of individual parameters and compensative effects of parameter changes, also known as collinearity. Both problems have gained substantial attention. As a remedy, most approaches aim to select an optimal subset of parameters that is both sufficiently sensitive and has lowest collinearity. The identifiable subset can be then estimated jointly with the remaining parameters assumed fixed. The determinant of the FIM and its least eigenvalue are used to measure optimality [7–10] of the selected set. Pairwise clustering has also been proposed to reduce the number of parameters [9]. A posteriori methods focus on finding identifiable parameters when experimental data are available. The likelihood surface around its maximum is then examined by means of the Hessian matrix [11, 12]. A statistical concept of profile likelihoods is particularly helpful [13] in this case. Identifiability analysis is closely related to experimental design. It has been used to show how the information content in experimental measurements can be maximised [13–16]. Despite useful methodological developments performing routine modelling tasks with a multi-parameter model still constitutes a substantial challenge. Here, we introduce a natural, universal and simple measure to quantify similarity between groups of model parameters. The measure links canonical correlation analysis (CCA) with Shannon’s mutual information (MI) and is called MI-CCA throughout the paper. Similarity between model parameters has been previously addressed (e.g. [9, 10, 17]). However a precise, statistically interpretable similarity measure has not been proposed. MI-CCA, when employed in a hierarchical clustering, provides statistically meaningful and precise information about mutual compensability of parameters. It can also be used as an assistance tool to validate parameters identifiability in experimental planning. Apart from its simplicity and rigorous statistical interpretation, the main advantage of our tool is that it can be applied to large models, for which other, well established, approaches are computationally infeasible. We demonstrate the power of our framework by analysis of the NF- *κ*B and MAPK signalling models. We find that highly similar parameters constitute groups consistent with the network topology. For the NF- *κ*B model we analyse the majority of published experimental protocols [18–26] and examine parameters identifiability. We show how the method can be used to guide further experiments.

## Methods

A typical model of biochemical dynamics describes how abundances of a set of *k* molecular entities, *y*=(*y*_1_,…,*y*_*q*_,…,*y*_*k*_), change with time *t*. Deterministically it is usually written as an ordinary differential equation (ODE)

(1) $$ \frac{dy}{dt}=F(y,\theta),  $$

where *F*() is a law that determines the temporal evolution of *y* and implicitly contains a control signal. The vector *θ*=(*θ*_1_,…,*θ*_*l*_) is a vector of model parameters. To numerically simulate the model, parameter values and initial condition, (*y*_1_(0),…,*y*_*k*_(0)), must be set. The method proposed in this paper is a priori in nature, therefore the parameter values and initial conditions are not inferred from data and must be assumed in advance based on the modellers knowledge.

Often only certain components of *y*, for instance first *q*, *y*^(*q*)^=(*y*_1_,…,*y*_*q*_), at specified times, (*t*_1_,…,*t*_*n*_), are of interest. These components, which may correspond to experimentally measured variables, are denoted here as *Y*=(*y*^(*q*)^(*t*_1_),…,*y*^(*q*)^(*t*_*n*_)).

### Conventional sensitivity analysis fails to capture collective interactions between model parameters

Sensitivity analysis provides a prediction how *Y* will change, *∂Y*, in response to small changes in a single parameter, *∂θ*_*i*_, or all parameters, *∂θ*=(*∂θ*_1_,…,*∂θ*_*l*_). If changes in parameters are small, the problem is solved by finding the derivative of a solution of the equation (1), *y*(*t*), with respect to the parameter $\theta _{i}, z_{i}(t)=\frac {\partial y(t)}{\partial \theta _{i}}$. This derivative can be easily calculated by solving another ODE (see Additional file 1). Evaluation of *z*_*i*_(*t*) at the times and components of interests defines the sensitivity vector $S_{i}=\left (z_{i}^{(q)}(t_{1}),\ldots,z_{i}^{(q)}(t_{n})\right)$ of the parameter *θ*_*i*_. The sensitivity vector describes the shift in *Y* in response to perturbation in the parameter *θ*_*i*_,*∂Y*=*S*_*i*_*∂θ*_*i*_. A collection of the sensitivity vectors for all *i*=1,…,*l* constitutes the sensitivity matrix *S*=(*S*_1_,…,*S*_*l*_), which summarises the change in *Y* in response to perturbation of all of the model parameters *∂Y*=*S∂θ*. The sensitivity matrix, *S*, is directly linked with the concept of Fisher information. Given that *Y* is observed with the Gaussian unit variance error the FIM can be written as (see Additional file 1)

(2) $$ FI(\theta)=S^{T} S.  $$

Therefore the FIM contains information regarding the size of a perturbation, $||\partial Y||=\sqrt {\partial \theta ^{T} FI(\theta) \partial \theta }$. The pairwise similarity between parameters, quantified as the cosine between the *S*_*i*_ and *S*_*j*_ vectors, is also given by elements of the FIM, $\cos (S_{i},S_{j})= {S_{i}^{T}} S_{j}/{|| S_{i} || || S_{j} ||}$. It is not clear, however, how the FIM can serve as a tool to analyse mutual relations between groups of parameters. Below we provide a rigorous and practical solution to this problem.

### Measuring similarity between parameters groups

**Fig. 1:**
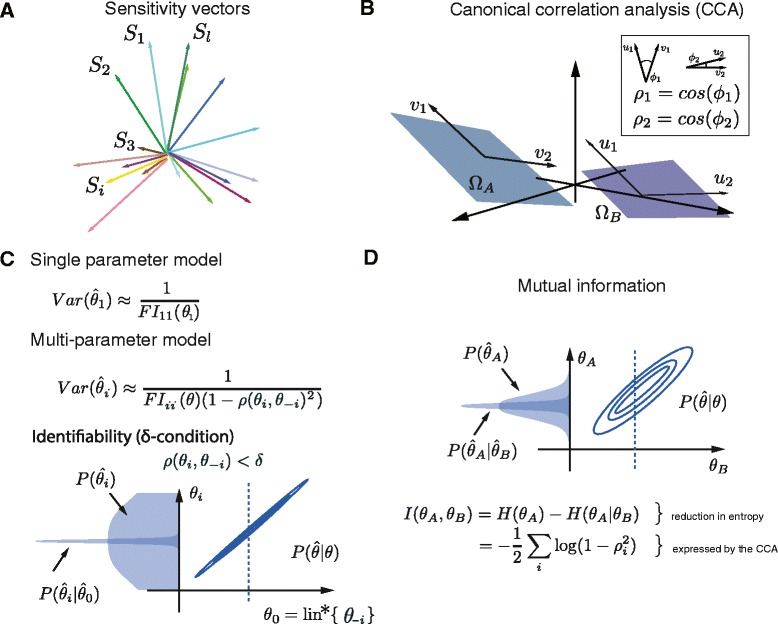
Canonical correlations and identifiability. **a** Illustrative view of the sensitivity vectors *S*_*i*_. **b** Conceptual illustration of the canonical correlations. Two subsets of sensitivity vectors represented as linear subspaces (planes *Ω*_*A*_ and *Ω*_*B*_). Canonical vectors on the planes are found to yield maximum cosine. In a two-dimensional subspace case, the second canonical vectors *u*_2_,*v*_2_ are required to be perpendicular to the first ones. **c** The introduced *δ*-condition requires that each parameter *θ*_*i*_ is correlated less than *δ* with the remaining parameters *θ*_−*i*_=(*θ*_1_,..,*θ*_*i*−1_,*θ*_*i*+1_,…,*θ*_*l*_). It can be interpreted in terms of how variance of the estimates changes when a single parameter and all model parameters are estimated. Parameter *θ*_0_ denotes the linear combination of *θ*_−*i*_ maximally correlated with *θ*_*i*_, i.e. *θ*_0_=lin^∗^{*θ*_−*i*_}. **d** Mutual information as a measure of similarity between two parameter sets *θ*_*A*_,*θ*_*B*_, which span linear subspaces *Ω*_*A*_,*Ω*_*B*_ interpreted in terms of the asymptotic posterior $P(\hat {\theta }|\theta)$

*Canonical correlations*. The canonical correlation analysis (CCA) is a simple extension of the Pearson correlation. With CCs it is possible to measure correlations between multidimensional covariates. We modify the well established definition to suit the considered context. Assume, we measure similarity between two subsets of parameters $\theta _{A}=\left \{\theta _{i_{1}},\ldots,\theta _{i_{a}} \right \}$ and $\theta _{B}=\left \{ \theta _{j_{1}},\ldots,\theta _{j_{b}} \right \}$ that correspond to the two subsets of sensitivity vectors, $\Omega _{A}=\left \{S_{i_{1}},\ldots,S_{i_{a}} \right \}$ and $\Omega _{B}=\left \{ S_{j_{1}},\ldots,S_{j_{b}} \right \}$. The latter can be interpreted as hyper-planes. CCs form a set of correlation coefficients defined recursively. The first CC, *ρ*_1_, is a maximal cosine between a linear combination, *u*_1_, in *Ω*_*A*_ and a linear combination, *v*_1_, in *Ω*_*B*_,*ρ*_1_= cos(*u*_1_,*v*_1_). Each next CC is found in the same way under the constraint that the next linear combination must be orthogonal to these found in the previous steps (see Additional file 1). Repeating the procedure *m*= min(*i*_*a*_,*j*_*b*_) times provides a set of CCs 1≥*ρ*_1_≥…≥*ρ*_*m*_≥0 (see Fig. 1a–b). The value of 1 indicates that there exists a linear combination of parameters in *θ*_*A*_ and *θ*_*B*_ having an identical impact, whereas 0 indicates existence of an orthogonal parameter combination. The CCs therefore provide an *m*-dimensional similarity measure between *θ*_*A*_ and *θ*_*B*_.

*Mutual information*. The above geometric view has a natural probabilistic interpretation that provides a natural, one-dimensional similarity measure. Assume, we estimate the parameter vector *θ* using the maximal likelihood estimate $\hat {\theta }$ (equivalently Bayesian posterior estimate) from data *X*=*Y*+*ξ*, where *ξ* is a measurement error. Asymptotically (for large number of independent copies of *X*, denoted here by *N*) the distribution of the estimate $\hat {\theta }$ given a true value *θ* is asymptotically multivariate normal

(3) $$ P(\hat{\theta}|\theta)\propto \exp(-\frac{1}{2N}(\hat{\theta}-\theta)FI(\theta)(\hat{\theta}-\theta)^{T}).  $$

Consider the entropy, $H(\hat {\theta }_{A})$, of the estimate $\hat {\theta }_{A} $, and the average conditional entropy of $\hat {\theta }_{A} $ given $\hat {\theta }_{B},H(\hat {\theta }_{A}|\hat {\theta }_{B})$. The reduction in entropy of $\hat {\theta }_{A}$ resulting from knowledge of $\hat {\theta }_{B} $ is given by Shannon’s mutual information between $\hat {\theta }_{A}$ and $\hat {\theta }_{B}$, denoted here by *I*(*θ*_*A*_,*θ*_*B*_). We propose to use *I*(*θ*_*A*_,*θ*_*B*_) as the natural measure of similarity. The more similar *θ*_*A*_ and *θ*_*B*_ are, the more knowing one will help in determining the value of the other. In Additional file 1 we show that the mutual information between estimates $\hat {\theta }_{A} $ and $\hat {\theta }_{B}$ and CCs are closely related

(4) $$ I(\theta_{A},\theta_{B})=H(\hat{\theta}_{A}) -H(\hat{\theta}_{A}|\hat{\theta}_{B})= -\frac{1}{m}{\sum_{i}^{m}} \log\left(1-{\rho_{i}^{2}}\right),  $$

where $H(\hat {\theta }_{A}|\hat {\theta }_{B})$ is the condition entropy of $\hat {\theta }_{A}$ given $\hat {\theta }_{B}$. The above measure, which throughout the paper is called MI-CCA, provides a novel and efficient way to quantify overall similarity between parameter groups via mutual information and CCs.

We use the constructed measures to propose a natural definition of parameters identifiability in the multi-parameter scenario.

### (*δ*,*ζ*)-identifiability

Conventionally, parameters of a statistical model *P*(*Y*|*θ*) are said to be identifiable if there exists a neighbourhood of *θ* such that for all parameter values in that neighbourhood *P*(*Y*|*θ*) represents a different distribution. Equivalently the FIM must have the full rank. This definition refers simultaneously to the entire vector of model parameters *θ*. The definition of [13] introduces a notion of practical non-identifiability by examining the flatness of the likelihood surface. We propose a novel definition of identifiability of individual parameters in multi-parameters models. It is widely recognised that lack of identifiability can arise from two sources: lack of sensitivity, or compensation of a parameter by remaining model parameters [7–10, 12, 27–30]. A definition that quantifies this intuition has been missing. Therefore, we propose a natural criterion of whether the parameter *θ*_*i*_ can be identified along with the remaining model parameters, *θ*_−*i*_. The parameter *θ*_*i*_ is said to be (*δ*,*ζ*)-identifiable if *ρ*(*θ*_*i*_,*θ*_−*i*_)<*δ* and ||*S*_*i*_||>*ζ*. Correlation *ρ* is used here in the canonical sense. If *θ*_*i*_ was estimated as a single parameter of the model *ζ*-condition requires its asymptotic variance to be smaller than 1/*ζ*. The *δ*-condition requires the parameter not to be correlated with any linear combination of the remaining parameters by more than *δ*. In variance terms, it translates into demanding that the variance does not increase by more than 1/(1−*δ*^2^) when the single parameter and multi-parameter scenarios are compared (Fig. 1c). The above definition is conceptually similar to the profile likelihood approach. However it uses asymptotic likelihood instead of actual likelihood and therefore does not require any numerical optimisation. Based on the FIM, solutions are given analytically by CCs. As a result identifiability can be determined for models of virtually any size. In practical applications values of *δ* and *ζ* must be selected. The above interpretation of *δ* and *ζ* values provides a theoretical ground to guide how these thresholds can be set. For instance, in the logarithmic parametrisation setting *ζ*=1 requires a parameter to be learned with at most an order of magnitude error. Parameter *δ* controls how the estimate’s variance increases when the parameter is estimated as a single parameter and jointly with remaining model parameters. Setting stricter values (lower *δ* and higher *ζ*) will result in lower variance of parameter estimates. Efficiency of the method enables the analysis to be performed for a range *δ* and *ζ* values that correspond to different levels of stringency. In the applications considered in this paper we used *ζ*=1 and *δ*=0.95. The latter corresponds to approximately 10-fold increase of variance (Fig. 1c). In Additional file 1 we use one of the analysed experiments to show that these thresholds provide results consistent with the profile likelihood approach. In general, profile likelihoods can also be used to validate method’s predictions as experimental data become available (see Sections 4.3 and 6.6 of the Additional file 1).

### Clustering reveals similarity structure and identifiability

**Fig. 2:**
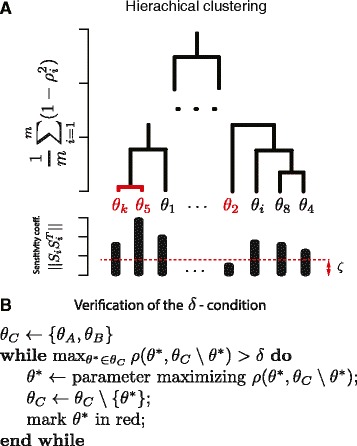
**a** Agglomerative hierarchical clustering of model parameters. **b** Verification of the *δ*-condition. Recursively, at each level, a pair of most similar clusters is merged into a single cluster and *δ*-condition is verified. Linkages between clusters, at each stage of clustering, are plotted at high $\frac {1}{m}{\sum \nolimits }_{i=1}^{m}(1-\rho ^{2})$, where *m* is the size of a new cluster, compared to a previous linkage. Identifiability results from violation of either of the *δ*-condition or *ζ*-condition therefore even parameters that have sensitivities above a threshold can be non-identifiable. Non-identifiable parameters are marked red

Using the constructed similarity measure we can meaningfully group model parameters. We provide a modification of the conventional hierarchical clustering algorithm. At each level of the hierarchy, clusters are created by merging clusters at the next lower level. At the lowest level, each cluster contains a single parameter. The pair chosen for merging consists of the two groups with the highest mutual information, *I*(*θ*_*A*_,*θ*_*B*_). When a new cluster is formed we verify if each of the parameters within the newly created cluster satisfies the *δ*-condition. The parameters of the clusters most correlated with the remaining parameters of the cluster are removed until all satisfy the *δ*-condition. We use average canonical correlation between the clusters, $\frac {1}{m}{\sum \nolimits }_{i=1}^{m}(1-\rho ^{2})$, which is normalised opposed to *I*(*θ*_*A*_,*θ*_*B*_), to determine the height of linkages. A set of identifiable parameters is not guaranteed to be maximal. Finding the maximal set would require testing each of the subsets of the parameter set, which is computationally infeasible. As the output of the algorithm, we obtain the visualisation of similarity structure and a set of identifiable parameters (see Fig. 2). The pseudocode describing the clustering algorithm in details is presented in Section 3 of the Additional file 1 and an R-implementation (Additional file 2) is available as an online supplement.

### Example: a simple gene expression model

To clarify the principles behind the method, we use a simplistic gene expression model. We assume that the process begins with the production of mRNA molecules at rate *k*_*r*_. Each mRNA molecule *r* may be independently translated into protein molecules at rate *k*_*p*_. Both mRNA and protein molecules are degraded at rates *γ*_*r*_ and *γ*_*p*_, respectively. Therefore, we have the vector of model parameters *θ*=(*k*_*r*_,*k*_*p*_,*γ*_*r*_,*γ*_*p*_) and ODEs presented in Figure 1A in Additional file 1. Consider the steady state $Y=\left (\frac {k_{r}}{\gamma _{r}}, \frac {k_{r} k_{p}}{\gamma _{r}\gamma _{p}}\right)$. We address the following questions: 1) Which model parameters are most similar?; 2) Which parameters are identifiable?; 3) What consequence does the similarity structure have for the model robustness?; 4) How can the steady state experiment be modified to reduce parameter correlations? The similarity of the parameters is determined entirely by the response of the model to changes in parameter values. The steady state formula implies that perturbations in *k*_*r*_ and *γ*_*r*_ have the same impact i.e. they increase or decrease the RNA and protein level. The same holds for perturbations in *k*_*p*_ and *γ*_*p*_. On the contrary, a perturbation in (*k*_*r*_,*γ*_*r*_) does not have the same impact as one in (*k*_*p*_,*γ*_*p*_). The first pair affects the level of both RNA and protein; the latter only the level of protein. This intuition is formalised and visualised by the method. The linkage between parameters *k*_*r*_,*γ*_*r*_ and *k*_*p*_,*γ*_*p*_ is plotted at zero height, and the non-identifiable parameters are marked red (Figure 1B in Additional file 1). Linkage between the pairs is at a non-zero height, as they are not entirely correlated. As for model robustness, the dendrogram depicts that mutually compensative perturbations occur within pairs (*k*_*r*_,*γ*_*r*_) and (*k*_*p*_,*γ*_*p*_). The analysis highlights the sources of non-identifiability and therefore helps to find experiments that render more parameters identifiable. For instance, in this example, pushing the initial condition *r*(*t*_0_),*p*(*t*_0_) above the steady state levels changes the model dynamics (Figure 1C in Additional file 1). The resulting exponential decay is not invariant with respect to parameter changes. As a result all parameters can be identified (Figure 1C in Additional file 1).

## Results

The NF- *κ*B pathway is one of the key components controlling the innate immune response. The model considered (see Additional file 1) was first proposed in [3] and further developed in [26]. For the simulations we have used parameter values and initial conditions introduced therein and reproduced in the Table 1 of the Additional file 1. The model represents a dynamic activation of NF- *κ*B induced genes in response to stimulation by a pro-inflammatory cytokine, TNF- *α*. It involves 39 parameters and 19 variables and encapsulates typical features of systems biology models. We address three questions: 1) What can we learn from the structure of parameter similarities? 2) Which parameters of the network can be estimated from the experiments published in the literature? 3) What experiments can be performed to increase the number of identifiable parameters?

**Fig. 3:**
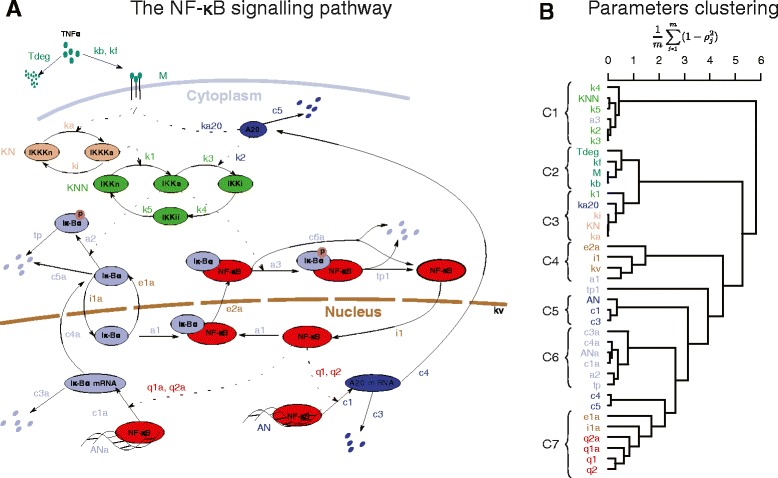
The NF *κ*B signalling pathway. **a** Schematic representation of the reactions involved. **b** Clustering of the model parameters reveals that parameters which characterise modules close in the network topology are generally more correlated than those far apart. Sensitivity vectors were calculated for a specific TNF- *α* stimulation to reflect physiological conditions (increase, plateau, decrease): see Figure 6B in Additional file 1

*Correspondence between parameter correlations and topology of the NF-κB system*. The dendrogram obtained for the NF- *κ*B system reveals that correlated parameters are grouped into clusters that largely correspond to the network structure (Fig. 3b). The cluster *C*1 contains parameters describing IKK kinase post-translational modifications and its interactions with the I *κBα*-NF- *κ*B complex; *C*2: TNF- *α* receptor activation and signalling; *C*3: IKKK kinase post-translational modifications and its interactions with A20 and IKK; *C*4: nuclear shuttling of NF- *κ*B and I *κBα* - NF- *κ*B binding; *C*4: A20 transcription and mRNA degradation; *C*6: I *κBα* transcription, translation, degradation and post-translational modifications *C*7: NF- *κ*B - DNA interactions and nuclear shuttling of I *κBα*.

The correspondence of the correlation structure with the network topology is one of the main findings of the paper. After that is explicitly stated it may seem intuitive. Although it provides relevant practical information, it has not been reported before. When aiming to change model dynamical response, parameters of various network modules should be manipulated rather than those within the same module. Regarding parameter inference, knowing a priori some parameters within various modules is more likely to help in estimating the remaining parameters than knowing the same number of parameters within a single module. The analogous conclusion holds for the system robustness. In the above analysis, we assumed that all model variables define model behaviour, i.e. *q*=*n*, and considered a response of the system to a physiological stimulation: gradual increase, plateau and gradual decrease of TNF- *α*. In a later subsection we present analogous observation for a MAPK signalling model. Earlier work of Huang *et al.* [31] reported similar fining using pairwise correlations. Moreover, the authors demonstrated that parameter correlations can be effectively used for systematic model reduction.

**Fig. 4:**
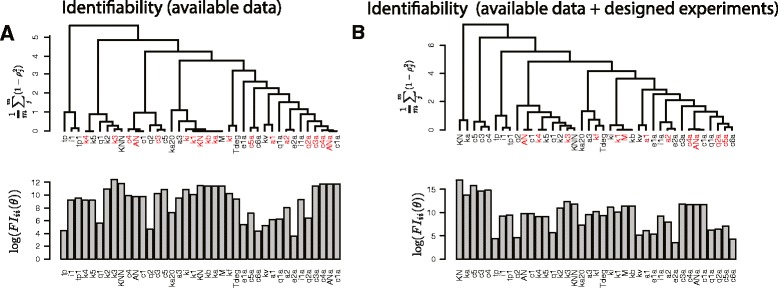
Identifiability study of the NF- *κ*B system. **a** Clustering results together with the identifiability analysis computed based on all the major published experiments. Non-identifiable parameters are marked in red. We used *δ*=0.95,*ζ*=1 to verify the identifiability condition. Sensitivity coefficients, i.e diagonal elements of the FIM, are shown below the dendrogram. **b** Clustering results as in (**a**) but for the published experiments together with the suggested experimental protocols

*Experiments examining the NF-κB dynamics jointly exhibit highly correlated parameters*. It is debatable how much data is needed to ensure parameters’ identifiability in systems biology models, and whether it is realistically achievable. Here we examined collectively all experiments reported in 9 papers [18–26] that contain rich data sets on the dynamics of the NF- *κ*B system. We asked which parameters of the NF- *κ*B model can be estimated from the published experiments (see Table 1 in Additional file 1). We found that 18 out of 39 model parameters cannot be estimated as they fail to satisfy the *δ*-condition (red parameters in Fig. 4a). The huge amount of literature available data, providing a comprehensive knowledge on the dynamics of the NF- *κ*B system, was not sufficient to ensure identifiability of all model parameters. The identifiability problem is widely reported. Here we demonstrate that it is not mitigated by a huge number of experiments performed to obtain insights other than values of kinetic rates. To draw our conclusions we have initially set *δ*=0.95 and *ζ*=1. As we used logarithmic parameterisation, i.e. log(*θ*_*i*_) instead of *θ*_*i*_ the latter corresponds to learning a parameter more accurately than with an order of magnitude error if the remaining model parameters were known. Value *δ*=0.95 requires the estimate’s variance not to increase by more than approximately 10 times when the single parameter and multi-parameter scenarios are compared. Thereafter we have verified that our main findings remain robust to assumptions regarding specific values of *δ* and *ζ* (Figure 3 in Additional file 1). We have also analysed how each of the analysed papers increased the number of identifiable parameters (Figure 2 in Additional file 1). Chronologically first two papers [18, 19], rendered 13 parameters identifiable. Subsequent 7 papers provided information to estimate 8 new parameters, which gives approximately 1 parameter per paper. This indicates that making more parameters identifiable requires specifically tailored experiments different to these performed to address conventional biological questions.

Given the size of the model analysed and the size of the data included in the aforementioned papers a posterior identifiability analysis would be hardly feasible. Identifiability studies available so far analyse single or small number of experiments. Importantly the dendrogram in Fig. 4a identifies which parameters are most correlated and therefore non-identifiable. This information can be effectively used to design experimental perturbations that decrease parameter correlations and enhance parameters identifiability.

*Tailored experiments can decrease parameter correlations and increase the number of identifiable parameters*. In order to find experiments that can provide information about non-identifiable parameters, we first randomly searched a space of potential new TNF- *α* stimulation time-profiles that together with available data would make new parameters identifiable. Details of considered protocols are presented in Additional file 1. We have assumed that only variables proven before to be measurable could be quantified. After having generated 1000 random TNF- *α* stimulation time-profiles we surprisingly found that none of the generated protocols can make more parameters to satisfy (*δ*,*ζ*)-condition. The underling cause is show in Figure 5 in Additional file 1: in all such protocols certain parameters have close to 1 correlation with the remaining parameters. This finding indicates that a successful strategy to obtain new identifiable parameters in multi-parameter models may require more careful design of new experiments. Correlation structure (Fig. 4a) revealed the underlying cause of non-identifiability and therefore we can select some of the highly correlated parameters to be estimated in additional experiments. We propose a small number of experiments that lead to identifiability of *ki*, *KN*, *ka*, *c*3,*c*4, and *c*3*a*. Here we describe how *ki*, *KN*, *ka* can be estimated whereas experiments to estimate *c*3,*c*4 and *c*3*a* are described in Additional file 1. Parameters *ki*, *KN*, *ka* and *ka*20 describe dynamics of phosphorylated IKKK (*y*_1_).

(5) $$ \dot{y}_{1}=ka\ y_{16}\ (KN-y_{1})\ ka20/(ka20+y_{9})-ki\ y_{1},  $$

where *y*_16_ and *y*_9_ denote activated TNF- *α* receptors and cytoplasmic A20 protein, respectively (see also equation (31) in Additional file 1). We assume phosphorylated IKKK, phosphorylated TNF- *α* receptors and cytoplasmic A20 protein can be measured by means of immunchemistry and we are able to evaluate the equation and compare it to a data. As identified by the dendrogram (Fig 4a), structure of the equation (5) also indicates that considered three parameters have very similar impact on *y*_1_. Figure 7A in Additional file 1 shows that indeed in a TNF- *α* stimulation experiment in wild type cells all parameters are highly correlated and non-identifiable. However, combining the dynamics in wild type cells, in A20 knockout cells and in A20 knockout cells with blocked phosphatase activity provides information to make *ki*,*KN* and *ka* identifiable (Figure 7C in Additional file 1). We verified that these identifiability predictions are correct using profile likelihood approach (Figure 7 B,D in Additional file 1). Identifiability also does not depend on specific parameter values used (Figure 7E in Additional file 1).

*Analysis of the MAPK signalling model*. In order to verify whether other biochemical models exhibit similar properties regarding correspondence between parameters similarity and network topology we have performed analysis of a MAPK signalling model [32]. The dendrogram of this model reflects the network topology (Figure 9 and 10 in Additional file 1). Our observations, therefore, might have a more general character. The model of [32] incorporates over 200 parameters and 100 equations. Computations required to plot dendrogram take several minutes on a standard desktop computer. The computational time scales with the cube of number of parameters. Therefore, the method can be applied to much larger models.

## Discussion and conclusions

The mutually compensative effects of parameters changes in mathematical models have gained substantial attention in recent years [1, 4, 5, 27, 28, 33]. Methods to better understand origins and consequences of parameter correlations have began to emerge. Particularly, authors of [7] defined identifiability of parameter subsets using the smallest eigenvalue of corresponding sub-matrices of the FIM. Selection of an identifiable set of parameters based on orthogonalisation of sensitivity vectors was proposed in [8, 10]. In [9, 10, 17] authors used pairwise correlations to better understand parametric sensitivity. In addition, the method introduced in [17] allows to detect existence of an explicit functional relationship between parameters but, in contrary to our method, it does not quantify the degree of collinearity. The existing methods are largely based on the determinant, the eigenvalues of the FIM or the pairwise correlations, and do not reveal the complexity of mutual relationships between parameters in multi-parameter models. Pairwise correlations cannot reflect similarity between groups of parameters. For instance, three parameters that have low pairwise correlations can be jointly non-identifiable. This is detected by CCA. MI-CCA allowed us to phrase intuitions about the impact of parameter correlations on parameter sensitivity and identifiability in a natural, statistically justified framework. In addition efficiency of the method makes it ideally suitable for large ODE models.

In the setting of this paper the mutual information *I*(*θ*_*A*_,*θ*_*B*_) is calculated based on the asymptotic posterior (3), which makes it exceptionally efficient to calculate in the local scenario. The concept however is very general and can be easily extended to the global case at the price of more intensive computations (see Additional file 1).

Apart from methodological development, the paper provides relevant insight into how experiments designed for purposes other than parameter estimation contribute to identifiability of model parameters. Non-identifiability problem may not be easily mitigated by collecting large number of measurements in experiments aimed at biological insight other than parameter estimation. Despite exceptionally rich data on the NF- *κ*B dynamics, a large fraction of model parameters remains non-identifiable. Experimental design strategies to be used in the multi-parameter scenario have not been developed yet. Systematic improvement of experimental design requires origins of non-identifiability to be pinpointed and removed. Our method constitutes a theoretically grounded approach to examine link between correlations and non-identifiability in a systematic way. Having a precise picture how correlations translate into non-identifiability allows targeted and rational design of further experiments. However it does not provide any automated or systematic approach to indicate a sequence of experiments leading to a full identifiable model. It only provides information to the modeller regarding sources of non-identifiability. It only helps to understand how non-identifiability arrises and provides guidelines whether considered experimental perturbations can remove detected correlations.

## Additional files

Additional file 1
**Supplementary information containing detailed description of the method and reported results.** (PDF 2467 kb)

Additional file 2
**Open source R implementation of the clustering algorithm is available at** http://sysbiosig.org/start/resources/ **.** (PDF 2.88 kb)
