# Supplementary material for: Clustering reveals limits of parameter identifiability in multi-parameter models of biochemical dynamics
Source: BMC Syst Biol. 2015 Sep 29;9:65. doi: 10.1186/s12918-015-0205-8 (PMC4587803; doi:10.1186/s12918-015-0205-8)
Supplement: Additional file 1 — Supplementary information containing detailed description of the method and reported results. (PDF 2467 kb) [file 12918_2015_205_MOESM1_ESM.pdf]

Supplementary Information

Clustering reveals limits of parameter identifiability in multi-parameter models of biochemical dynamics

Karol Nienałtowski[1], Michał Włodarczyk[2], Tomasz Lipniacki[1], Michał Komorowski[1]

1. Institute of Fundamental Technological Research, Polish Academy of Sciences, Warsaw, Poland
2. Faculty of Mathematics Informatics and Mechanics, University of Warsaw, Poland

This is the supplementary information for the paper *Clustering reveals limits of parameter identifiability in multi-parameter models of biochemical dynamics* models which is henceforth referred to as the main paper (MP).

1 Background

In the first section we provide details of conventional modelling techniques that we incorporate in our framework.

1.1 Calculating sensitivities of ODE models

The methodology developed in this paper can in principle be applied to analyse any ordinary differential equation model. Precisely, we consider

$$\frac{dy}{dt} = F(y, \theta), \tag{1}$$

where $y = (y_1, ..., y_k) \in \mathbb{R}^k$ is a system's state; $F()$ is a law, which determines temporal evolution of y; and $\theta = (\theta_1 \ldots, \theta_l) \in \theta \subset \mathbb{R}^l$ is a vector of model parameters. For simplicity, instead of writing solution of (1) as a function of the time, t; an initial condition, y_0; and the parameters, θ we write simply $y(t)$. Without loss of generality, we assume that the first q components of y denoted by $y^{(q)} = (y_1, ..., y_q)$ contain model variables that are of interest. These components evaluated at specified times are denoted by $Y = (y^{(q)}(t_1), ..., y^{(q)}(t_n))$. The relationship between the mechanistic model (1) and experimental observations, X, is defined as

$$X = (y_q(t_1) + \epsilon_1, \ldots, y_q(t_n) + \epsilon_n), \tag{2}$$

where ϵ_i is a multivariate measurement noise. In the examples studied, without loss of generality, we assume the noise to be multivariate normal (MVN), $\epsilon_i \sim MVN(0, D_{\epsilon_i})$ with D_{ϵ_i}, being a diagonal matrix of measurement variances. In this setting $X \sim MVN(Y, D)$, where D is a matrix that contains matrices D_i on its diagonal.
The derivative of solution of the equation (1), $y(t)$, with respect to the parameter θ_i, $z_i(t) = \frac{\partial y(t)}{\partial \theta_i}$ is described by an another ODE [1]

$$\frac{dz_i(t)}{dt} = \nabla_y F(y(t), \theta) z_i(t) + \frac{\partial F(y(t), \theta)}{\partial \theta_i}, \tag{3}$$

where $\nabla_y F(y(t), \theta)$ is the Jacobian matrix of $F()$ with respect to y. Evaluation of $z_i(t)$ at the times and components of interests defines the sensitivity vector $S_i = \left(z^{(q)}(t_1), ..., z^{(q)}(t_n)\right)$ of the parameter θ_i. A collection of the sensitivity vectors for all $i = 1, ..., l$ constitutes the sensitivity matrix $S = (S_1, ..., S_l)$.
The Fisher information matrix (FIM) of the MVN with density $P(X|\theta)$ defined as

$$FI_{i,j}(\theta) = E\left(\frac{\partial \log(P(X|\theta))}{\partial \theta_i} \frac{\partial \log(P(X|\theta))}{\partial \theta_j}\right)$$

can be written in terms of sensitivity vectors [2]

$$FI(\theta) = \frac{1}{\sigma^2} S^T D^{-1} S. \tag{4}$$

For convenience and without loss of generality in the paper we assumed $D = I$, where I is the identity matrix.

1.2 Logarithmic parametrisation

Throughout the paper we parametrise models in terms of logarithms of their parameters, $\log(\theta_i)$. For notational convenience we write simply θ_i instead of $\log(\theta_i)$.

1.3 Shannon Mutual Information

In the paper we use mutual information to measure similarity between groups of parameters. Parameters estimates are treated as random variables. First we introduce definition of mutual information and consider general random variables U and V having a joint probability distribution $P(U, V)$ and marginal distributions $P(U)$, $P(V)$. Using the Shannon entropy [3], uncertainty associated with a random variable is given as

$$H(U) = -\int P(U) \log(P(U)) dU. \tag{5}$$

Analogously the uncertainty of U conditioned on a given value of $V = v$ is given by conditional entropy

$$H_v(U) = -\int P(U|V = v)\log(P(U|V = v))dU. \tag{6}$$

Averaging over all possible values of V we have average conditional entropy

$$H(U|V) = -\int H_v(U)\log(P(v))dv. \tag{7}$$

Using the above notions the average reduction in entropy of U resulting from knowing V is given by the Shannon mutual information

$$I(U,V) = H(U) - H(U|V). \tag{8}$$

2 Mutual information - canonical correlation analysis (MI-CCA)

Below we explain calculation of $I(\theta_A, \theta_B)$ using CCs directly from Fisher information matrix. Assume, data $X^{(i)}$, $i = 1, ..., N$ are independent measurements of Y for given parameters θ. Under certain general assumptions the distribution of the maximum likelihood estimator or equivalently maximum a posteriori estimator $\hat{\theta}$ is asymptotically multivariate normal [4]

$$P(\hat{\theta}|\theta) \propto \exp(-\frac{1}{2N}(\hat{\theta} - \theta)FI(\theta)(\hat{\theta} - \theta)^T). \tag{9}$$

See conditions A1-A9 in [5] or Bernstein-von Mises Theorem in [6] for details.
Suppose $\theta = (\theta_A, \theta_B)$, $\theta_A = (\theta_1, ..., \theta_m)$ and $\theta_B = (\theta_{m+1}, ..., \theta_l)$. Given the equation (9) we can divide the Fisher information matrix and the covariance matrix $\Sigma = FI^{-1}$ into components corresponding to θ_A and θ_B

$$\Sigma = \begin{pmatrix} \Sigma_{AA} & \Sigma_{AB} \\ \Sigma_{BA} & \Sigma_{BB} \end{pmatrix} \tag{10}$$

and

$$FI = \begin{pmatrix} FI_{AA} & FI_{AB} \\ FI_{BA} & FI_{BB} \end{pmatrix}. \tag{11}$$

The mutual information formula for the multivariate normal can be easily computed using one of the following well known formulae [7]

$$I(\theta_A, \theta_B) = -\frac{1}{2}\log\left(\frac{|\Sigma|}{|\Sigma_A||\Sigma_B|}\right) \tag{12}$$

or

$$I(\theta_A, \theta_B) = -\frac{1}{2}\sum_{j=1}^{k} log(1 - (\rho_j^M)^2) \tag{13}$$

where $|\cdot|$ denotes the determinant of a matrix and ρ_i^M are canonical correlations calculated from Σ. Precisely

$$\rho_i^M = \max_{w_A^i, w_B^i} \left(\frac{{w_A^i}^T \Sigma_{AB} w_B^i}{\sqrt{{w_A^i}^T \Sigma_{AA} w_A^i {w_B^i}^T \Sigma_{BB} w_B^i}} \right) \tag{14}$$

subject to $(i \neq j)$

$${w_A^i}^T \Sigma_{AA} w_A^j = 0,$$
$${w_B^i}^T \Sigma_{BB} w_B^j = 0,$$

and

$${w_A^i}^T \Sigma_{AB} w_B^j = 0$$

for $i = \min(m, l-m)$ and $j < i$. The formulae (12) and (13) have however two strong disadvantages: involve inversion of the FIM, and requires division by the matrix determinant, which can be close to zero.

Therefore we propose a new approach that avoids these difficulties. Specifically we show that a formula based on canonical correlations calculated directly from the FIM instead of the covariance matrix also holds

$$I(\theta_A, \theta_B) = -\frac{1}{2}\sum_{j=1}^{k} log(1 - \rho_j^2), \tag{15}$$

where ρ_i are canonical correlations calculated directly from $FI(\theta)$. These are defined as

$$\rho_i = \max_{w_A^i, w_B^i} \left(\frac{{w_A^i}^T FI_{AB} w_B^i}{\sqrt{{w_A^i}^T FI_{AA} w_A^i {w_B^i}^T FI_{BB} w_B^i}} \right) \tag{16}$$

subject to $(i \neq j)$

$${w_A^i}^T FI_{AA} w_A^j = 0,$$
$${w_B^i}^T FI_{BB} w_B^j = 0,$$

and

$${w_A^i}^T FI_{AB} w_B^j = 0,$$

for $i = \min(m, l-m)$ and $j < i$.

Proof
It is sufficient to prove that
$$\frac{|\Sigma|}{|\Sigma_A||\Sigma_B|} = \frac{|FI|}{|FI_A||FI_B|}. \tag{17}$$
Recall that
$$\begin{pmatrix} \Sigma_{AA} & \Sigma_{AB} \\ \Sigma_{BA} & \Sigma_{BB} \end{pmatrix}^{-1} = \begin{pmatrix} FI_{AA} & FI_{AB} \\ FI_{BA} & FI_{BB} \end{pmatrix} \tag{18}$$
As Σ is positively defined then $|\Sigma_{AA}| > 0$ and $|\Sigma_{BB}| > 0$. In particular Σ_{AA} and Σ_{BB} are nonsingular and we can take advantage of the formula for determinant of a block matrix

$$|\Sigma| = |\Sigma_{AA}||\Sigma_{BB} - \Sigma_{BA}\Sigma_{AA}^{-1}\Sigma_{AB}| \tag{19}$$

$$|\Sigma| = |\Sigma_{BB}||\Sigma_{AA} - \Sigma_{AB}\Sigma_{BB}^{-1}\Sigma_{BA}|.$$

First observation is that matrices $\Sigma_{BB} - \Sigma_{BA}\Sigma_{AA}^{-1}\Sigma_{AB}$ and $\Sigma_{AA} - \Sigma_{AB}\Sigma_{BB}^{-1}\Sigma_{BA}$ have nonzero determinants so they are invertible. This allows us to use formula for block-wise inversion

$$\begin{pmatrix} \Sigma_{AA} & \Sigma_{AB} \\ \Sigma_{BA} & \Sigma_{BB} \end{pmatrix}^{-1} = \begin{pmatrix} (\Sigma_{AA} - \Sigma_{AB}\Sigma_{BB}^{-1}\Sigma_{BA})^{-1} & \cdots \\ \cdots & (\Sigma_{BB} - \Sigma_{BA}\Sigma_{AA}^{-1}\Sigma_{AB})^{-1} \end{pmatrix} \tag{20}$$

Combining this with equation (18) we obtain

$$FI_{AA} = (\Sigma_{AA} - \Sigma_{AB}\Sigma_{BB}^{-1}\Sigma_{BA})^{-1} \tag{21}$$

$$FI_{BB} = (\Sigma_{BB} - \Sigma_{BA}\Sigma_{AA}^{-1}\Sigma_{AB})^{-1}$$

Next, we use formula (19) to observe that

$$|\Sigma| = \frac{|\Sigma_{AA}|}{|FI_{BB}|} = \frac{|\Sigma_{BB}|}{|FI_{AA}|} \tag{22}$$

After short transformations and replacing one $|\Sigma|$ with $|FI|^{-1}$ we finally get

$$\frac{|\Sigma|}{|\Sigma_{AA}||\Sigma_{BB}|} = \frac{|FI|}{|FI_{AA}||FI_{BB}|}. \tag{23}$$

3 Details of the clustering algorithm

The developed clustering algorithm is summarised as follows

calculate FIM;
$clusters \leftarrow \{\{\theta_1\}, \{\theta_2\} \dots \{\theta_k\}\}$ such that $FIM_{ii}(\theta) > \zeta$
for all $A \in clusters$ **do**
  $height(A) \leftarrow 0$;
end for
while $|clusters| > 1$ **do**
  for all $A, B \in clusters, A \neq B$ **do**
    calculate $I(A, B)$ using formula (15);
  end for
  $(A, B) \leftarrow$ pair of clusters maximizing $I(A, B)$;
  $(\rho_i)_{i=1}^{m} \leftarrow$ canonical correlation between A and B;
  create new node C representing the union of A and B;
  $height(C) \leftarrow max(height(A), height(B)) + \frac{1}{m}\sum_{i=1}^{m}(1 - \rho_i^2)$;
  while $\max_{\theta^* \in C} \rho(\theta^*, C \setminus \theta^*) > \delta$ **do**
    $\theta^* \leftarrow$ parameter maximizing $\rho(\theta^*, C \setminus \theta^*)$;
    $C \leftarrow C \setminus \{\theta^*\}$;
    mark θ^* in red;
  end while
  $clusters \leftarrow clusters - A - B + C$;
end while

Non-identifiable parameters are marked in red.

4 Interpreting (δ, ζ)-identifiability

As discussed in the subsection 2.3 of the MP the parameter θ_i is said to be (δ, ζ)-identifiable if $\rho(\theta_i, \theta_{-i}) < \delta$ and $||S_i|| > \zeta$. We purposely require the parameter to satisfy two independent conditions. The ζ-condition requires the parameter to have individual sensitivity above a threshold. The δ-condition requires the parameter not to be correlated with remaining parameters above the threshold. Such a definition is constructed to ensure that repeating the same experiment several times cannot make non-identifiable parameters identifiable and to determine which very parameters are not identifiable. In addition our focus on having non-correlated parameters results from advantages of manipulating models with a non-correlated parameters. These advantages are widely discussed in the literature [1, 8–11] .

In our approach, incorporating additional experiments that yield highly correlated parameters may decreasing the number of identifiable parameters. For illustration consider a model with two parameters $\theta = (\theta_1, \theta_2)$. Assume that the parameters can be informed by two experiments yielding the following FIMs

$$FI^{(1)} = \begin{pmatrix} 1 & c \\ c & 1 \end{pmatrix} \qquad FI^{(2)} = \begin{pmatrix} 1 & d \\ d & 1 \end{pmatrix} \tag{24}$$

Given independence of the two experiments the joint experiment results with the FIM

$$FI^{(1,\ 2)} = \begin{pmatrix} 2 & c+d \\ c+d & 2 \end{pmatrix}. \tag{25}$$

Comparing correlation in the three scenarios, denoted here by $\rho^{(1)}$, $\rho^{(2)}$, $\rho^{(1,2)}$, we have that correlation is averaged over experiments

$$\rho^{(1)} = c, \qquad \rho^{(2)} = d, \qquad \rho^{(1,2)} = \frac{1}{2}(c+d). \tag{26}$$

Therefore adding "bad" i.e. highly correlated data (experiment) may cause parameters fail to satisfy δ-condition. It may seem an undesirable property. From practical point of view in many cases it is however desirable. When performing inference in multi-parameter models the main difficulty arrises from the likelihood surface having the shape of a long thin ellipsoid [8]. This makes the search in parameter space computationally demanding. Our approach, tailored to deal with multi-parameter models, is aimed to recognise this difficulty.

4.1 Relationship of (δ, ζ)-identifiability to "alphabetical" experimental design criteria

The criterium of (δ, ζ)-identifiability is different from the conventional Fisher information based approaches known as alphabetic optimality (A-optimality, D-optimality, E-optimality etc.), which maximise overall information about model parameters [12]. These criteria do not serve to determine identifiability of individual parameter. We are focused on maximising the number of individual parameters. In addition, as opposed to our method, conventional approaches of alphabetic optimality predict increase of information as the same experiment is repeated.

4.2 Low pairwise correlations do not ensure identifiability

Measuring parameters similarity using MI-CCA has a qualitative advantage over pairwise correlations. Parameters that have low pairwise correlations may be non-identifiable and

have CC equal to 1. This can be easily exemplified by the following FIM with respect to parameters $\theta_1, \theta_2, \theta_3$

$$FI = \begin{pmatrix} 1 & 0 & \frac{\sqrt{2}}{2} \\ 0 & 1 & \frac{\sqrt{2}}{2} \\ \frac{\sqrt{2}}{2} & \frac{\sqrt{2}}{2} & 1 \end{pmatrix}. \qquad (27)$$

Straightforward calculation shows that we have the following pairwise correlations

$$\rho(\theta_1, \theta_2) = 0, \qquad \rho(\theta_1, \theta_3) = \rho(\theta_2, \theta_3) = \sqrt{2}/2.$$

However, CCA detects that θ_3 is a linear combination of θ_1 and θ_2 as

$$\rho((\theta_1, \theta_2); \theta_3) = 0.$$

4.3 Relationship of (δ, ζ)-identifiability to profile likelihoods based identifiability analysis

The concept of (δ, ζ)-identifiability is related to a concept of identifiability defined based on profile likelihoods [13, 14]. Our framework is complementary and should be applied in different scenarios. To demonstrate the link between the two approaches we introduce a standard statistical notation. Assume as before, data X have a distribution with a density $P(X|\theta)$. Identical independent copies of X are denoted by X_k. We also introduce: log-density $l(X|\theta) = \log(P(X|\theta))$; expected log-density $L(\theta) = E_{\theta^*}(l(X|\theta))$, where $E_{\theta^*}()$ denotes expected value with respect to $P(X|\theta^*)$; log-likelihood $L_n(\theta) = \sum_{k=1}^{N} l(X_i|\theta)$.
Log-profile likelihood, $PL_n(\cdot)$, of a parameter θ_i estimated along with parameters $\theta_{-i} = \theta \backslash \{\theta_i\}$ is conventionally defined as [13, 15, 16]

$$PL_n(\theta_i) = \max_{\theta_{-i}} \left(L_n(\theta)\right). \qquad (28)$$

We consider the second order Taylor expansion of $L(\theta)$ around a true value θ^*

$$L(\theta) = L(\theta^*) - \frac{1}{2} \left(\theta - \theta^*\right)^T FI(\theta^*) \left(\theta - \theta^*\right). \qquad (29)$$

The first order term is missing as $L(\theta)$ has the maximum for $\theta = \theta^*$ and therefore $\nabla_\theta L(\theta^*) = 0$. The second term is by definition described by Fisher information. The function defined below we call asymptotic profile likelihood (APL)

$$APL(\theta_i) = \max_{\theta_{-i}} \left(L(\theta)\right). \qquad (30)$$

It is well known that assuming general regularity conditions, we have convergence $L_n(\theta) \to L(\theta)$ for each θ and therefore also convergence $PL_n(\theta) \to APL(\theta)$ as $n \to \infty$.
Consider the following transformation

$$APL(\theta_i) = \max_{\theta_{-i}} \left(L(\theta)\right) = L(\theta^*) - \frac{1}{2} \min_{\theta_{-i}} \left(\frac{1}{2} \left(\theta - \theta^*\right)^T FI(\theta^*) \left(\theta - \theta^*\right) \right)$$

Direct calculation (see proof below) shows that

$$\min_{\theta_{-i}} \left(\left(\theta - \theta^*\right)^T FI(\theta^*) \left(\theta - \theta^*\right) \right) = FI_{ii}(\theta^*) \left(1 - \rho^2\right) \left(\theta_i - \theta_i^*\right)^2,$$

where ρ is the CC between θ_i and θ_{-i}.

Therefore we have the formula that allows us to understand the link between (δ, ζ)-identifiability and PL based identifiability

$$APL(\theta_i) = L(\theta^*) - \frac{1}{2} FI_{ii}(\theta^*) \left(1 - \rho^2\right) \left(\theta_i - \theta_i^*\right)^2.$$

Consider curvature κ of APL measured as the signless, second order derivative with respect to θ_i

$$\kappa = \left| \frac{\partial^2 APL(\theta_i)}{\partial \theta_i^2} \right| = FI_{ii}(\theta^*) \left(1 - \rho^2\right).$$

The assumption of (δ, ζ)-identifiability imposes conditions on the curvature of APL. The contribution resulting from FI_{ii} must be greater than ζ and the contribution coming from correlation must be greater than $(1 - \delta^2)$. We purposefully neglect the actual value of FI_{ii} and verify only if it is above a threshold, as it can be artificially inflated by scaling of the parameter or repeating the same experiment several times. The correlation ρ is affected by neither of these factors.

The identifiability based on profile likelihoods [13] is determined by confidence intervals $\{\theta_i : |PL(\theta_i) - PL(\hat{\theta}_i)| < \Delta\}$, where $\hat{\theta}$ is the argument maximising $L_n(\theta)$, and Δ is a constant selected based on χ^2 statistics. If confidence interval extends infinitely parameter is non-identifiable.

The two methods are applicable in different practical situations. If data X is available and $L_n(\theta)$ can be calculated profile likelihood is the superior method. Our method, however, is tailored to deal with a situation where only $L(\theta)$ is available, whereas $L_n(\theta)$ is not. It is the case in a number of scenarios. For instance, if data X is not (yet) available or $L_n(\theta)$

cannot be evaluated analysing APL is the best one can do. As we demonstrate clustering provides informative tool to explain the curvature of APLs, which cannot be delivered by available methods. In the case of our meta-analysis of the NF-κB experiments calculating $L_n(\theta)$ would hardly be possible due to reasons related to availability / comparability of data as well as computational efficiency.

Proposition

The minimum $\min_{\theta_{-i}} \left((\theta - \theta^*)^T FI(\theta^*) (\theta - \theta^*) \right)$ can be expressed via CC using the following formula

$$\min_{\theta_{-i}} \left((\theta - \theta^*)^T FI(\theta^*) (\theta - \theta^*) \right) = FI_{ii}(\theta^*) \left(1 - \rho^2\right) (\theta_i - \theta_i^*)^2.$$

Proof

Define $G(\theta_{-i}|\theta_i) = d\theta^T FI(\theta^*) d\theta$, where $d\theta = \theta - \theta^*$. Differentiation with respect to θ_{-i} given θ_i shows that the minimum is achieved for

$$d\theta_{-i} = -FI_{-i,-i}(\theta^*)^{-1} F_{-i,i}(\theta^*) d\theta_i,$$

where lower indices denote corresponding elements of the FIM. We have therefore

$$\min_{\theta_{-i}} G(\theta_{-i}|\theta_i) = d\theta_i^2 FI_{ii}(\theta^*) \left(1 - \frac{FI_{-i,i}(\theta^*) FI_{-i,-i}(\theta^*)^{-1} FI(\theta^*)_{i,-i}}{FI_{i,i}(\theta^*)}\right)$$

Calculating CC, ρ, between θ_i and θ_{-i} we obtain that

$$\rho = \frac{FI_{-i,i}(\theta^*) FI_{-i,-i}(\theta^*)^{-1} FI(\theta^*)_{i,-i}}{\sqrt{FI_{i,i}(\theta^*) FI_{-i,i}(\theta^*) FI_{-i,-i}(\theta^*)^{-1} FI(\theta^*)_{i,-i}}}.$$

Hence,

$$\min_{\theta_{-i}} G(\theta_{-i}|\theta_i) = d\theta_i^2 FI_{ii}(\theta^*)(1 - \rho^2).$$

5 Gene expression model

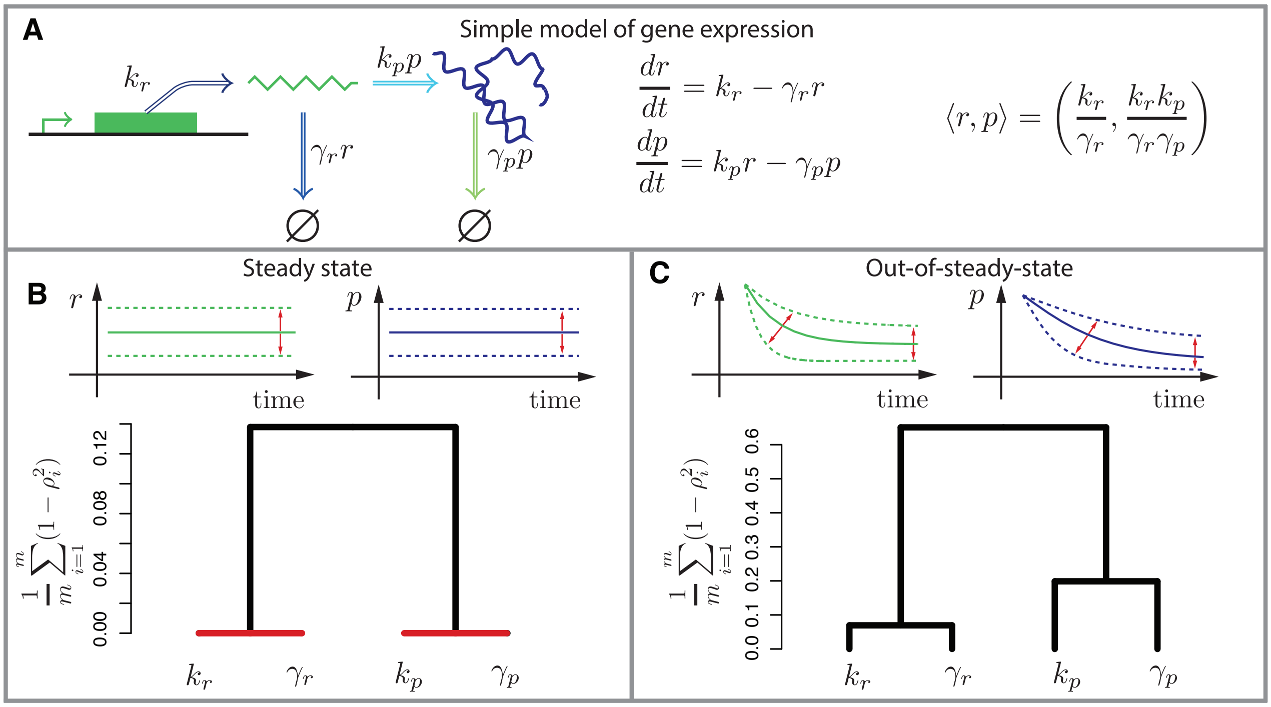

Figure 1: Analysis of the simple model of gene expression. (**A**) Model reactions, corresponding ODEs and the steady state. (**B**) Illustrative analysis of the steady state dependence on parameters. (**C**) Analysis of the system out-of-steady-state, the initial condition was increased 30-fold compared to the steady state. See text for a discussion. The dendrograms were generated using $k_r = 100$, $k_p = 2$, $\gamma_r = 1.2$, $\gamma_p = 0.8$.

6 Analysis of the NF-κB system

The NF-κB dynamical model together with used parameter values are taken directly from [17] and reader is referred to the supplementary information of that paper for more details. Below we reproduce equations of the model and its parameter values (Table 1).

6.1 Model equations

$$
\begin{aligned}
\text{active IKKK kinase} \quad \frac{y_1}{dt} &= ka\ y_{16}\ (KN - y_1)\ ka20/(ka20 + y_9) - ki\ y_1 \qquad (31)\\
\text{neutral IKK (IKKn)} \quad \frac{y_2}{dt} &= -y_1^2\ k1\ y_2 + k5\ (KNN - y_2 - y_3 - y_4)\\
\text{free active IKK (IKKa)} \quad \frac{y_3}{dt} &= y_1^2\ k1\ y_2 - k3\ y_3\ (k2 + y_9)/k2\\
\text{inactive IKK (IKKi)} \quad \frac{y_4}{dt} &= k3\ y_3\ (k2 + y_9)/k2 - k4\ y_4\\
\text{Phospo-I}\kappa\text{B}\alpha\text{ cytoplasmic} \quad \frac{y_5}{dt} &= a2\ y_3\ y_{11} - tp\ y_5\\
\text{cytoplasmic (phospho-I}\kappa\text{B}\alpha\text{—NF}\kappa\text{B)} \quad \frac{y_6}{dt} &= a3\ y_3\ y_{14} - tp1\ y_6\\
\text{free cytoplasmic NF}\kappa\text{B} \quad \frac{y_7}{dt} &= c6a\ y_{14} - a1\ y_7\ y_{11} + tp1\ y_6 - i1\ y_7\\
\text{free nuclear NF}\kappa\text{B} \quad \frac{y_8}{dt} &= i1\ y_7 - a1\ kv\ y_{12}\ y_8\\
\text{cytoplasmic A20} \quad \frac{y_9}{dt} &= c4\ y_{10} - c5\ y_9\\
\text{A20 transcript} \quad \frac{y_{10}}{dt} &= c1\ y_{17} - c3\ y_{10}\\
\text{free cytoplasmic I}\kappa\text{B}\alpha \quad \frac{y_{11}}{dt} &= -a2\ y_3\ y_{11} - a1\ y_{11}\ y_7 + c4a\ y_{13} - c5a\ y_{11} - i1a\ y_{11} + e1a\ y_{12}\\
\text{free nuclear I}\kappa\text{B}\alpha \quad \frac{y_{12}}{dt} &= -a1\ kv\ y_{12}\ y_8 + i1a\ y_{11} - e1a\ y_{12}\\
\text{I}\kappa\text{B}\alpha\text{ transcript} \quad \frac{y_{13}}{dt} &= c1a\ y_{18} - c3a\ y_{13}\\
\text{cytoplasmic (I}\kappa\text{Ba—NF}\kappa\text{B) complex} \quad \frac{y_{14}}{dt} &= a1\ y_{11}\ y_7 - c6a\ y_{14} - a3\ y_3\ y_{14} + e2a\ y_{15}\\
\text{nuclear (I}\kappa\text{Ba—NF}\kappa\text{B) complex} \quad \frac{y_{15}}{dt} &= a1\ kv\ y_{12}\ y_8 - e2a\ y_{15}\\
\text{active receptors} \quad \frac{y_{16}}{dt} &= kb\ y_{19}\ (M - y_{16}) - kf\ y_{16}\\
\text{A20 gene state} \quad \frac{y_{17}}{dt} &= q1\ y_8\ (AN - y_{17}) - q2\ y_{12}\ y_{17}\\
\text{I}\kappa\text{B}\alpha\text{ gene state} \quad \frac{y_{18}}{dt} &= q1a\ y_8\ (ANa - y_{18}) - q2a\ y_{12}\ y_{18}\\
\text{extracellular TNF-}\alpha \quad \frac{y_{19}}{dt} &= -\ Tdeg\ y_{19}
\end{aligned}
$$

6.2 Variable normalisation

In the entire analysis of the NF-κB system we normalised model variables by squared root of their maxima. We also assumed that the normalised variables were measured with unit variance normal measurement error. Precisely

$$X = (Y_{i_1}/\sqrt{\max(Y_{i_1})} + \epsilon_{i_1}, ..., Y_{i_q}/\sqrt{\max(Y_{i_q})} + \epsilon_{i_q}),$$

where $\epsilon_{i_j} \sim MVN(0, I)$, where I is an identity matrix. Indices i_j select for variables that are of interest in the context of the presented examples. These two assumptions are equivalent to assume that each Y_{i_j} is measured with the variance equal to the $\max(Y_{i_j})$.

6.3 Available experimental data - identifiability analysis

In the Table 2 we summarise experiments presented in the 9 papers [17–25] used to study identifiability of the NF-κB model. We simplistically assumed that all experiments were comparable and measurements across experiments were taken in the same units equivalent to those used in the model (31). We used algorithm (3) to find out the number of identifiable parameters using the (δ, ζ)-identifiability criterium. We set $\delta = 0.95$ and $\zeta = 1$. Starting from the first listed experiment we iteratively added subsequent experiments that maximised the number of identifiable parameters.
We repeated the procedure using experiments published in each of the papers and prior to its publication. Obtained cumulative number of identifiable parameters is presented in Figure 2. The dendrogram with 21 identifiable parameters for the total of available data is presented in Figure 4A in MP.

6.4 Dependance of the results on δ and ζ values

In order to verify how the identifiability predictions depend on choice of values of δ and ζ we have plotted the number of identifiable parameters as a function of δ and ζ in Figure 3. All parameters are well above considered ζ thresholds therefore the number of identifiable parameters does not change as ζ is varied. The number of identifiable parameters increases as higher correlation can be accepted and reaches the total number of model parameters (39) at $\delta = 1$. The number of identifiable parameters is sensitive to δ. However the main conclusions of our analysis that the rich set of available experiments yields highly correlated parameters holds for all values of δ smaller the one. The correlations are moderately decreased by the proposed experiments.

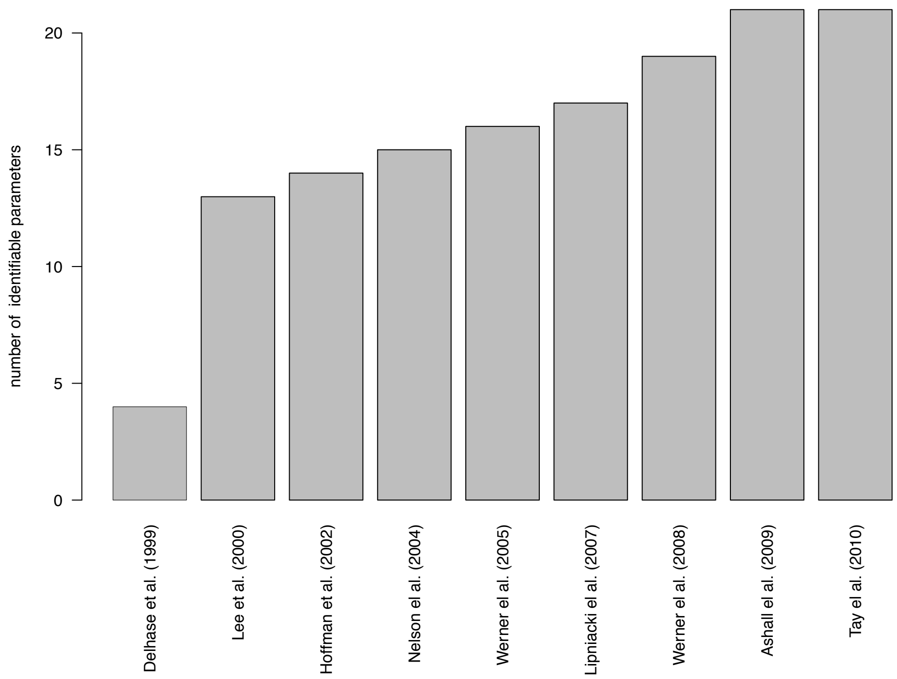

Figure 2: The number of (δ, ζ)-identifiable parameters in the NF-κB model using experiments published in the indicated paper and these available prior to its publication. Parameters $\delta = 0.95$ and $\zeta = 1$ were used.

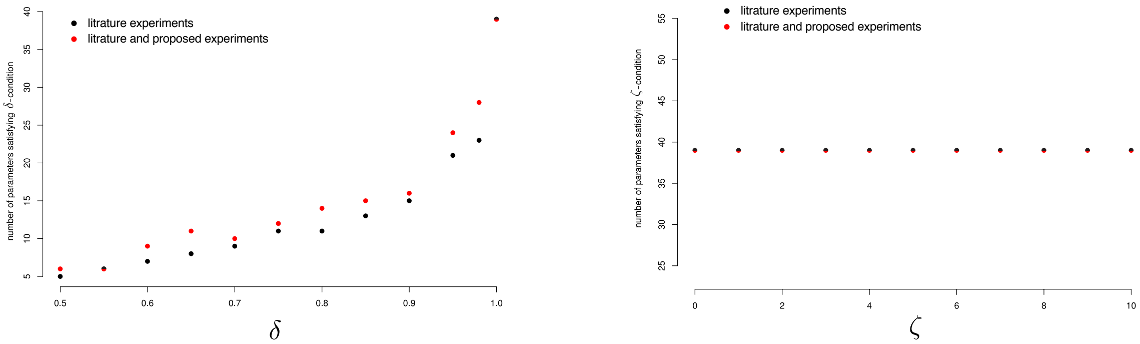

Figure 3: Number of parameters that satisfy the (δ, ζ)-condition as a function of δ (left) and as a function ζ (right), for literature experiments (black) and literature experiments together with proposed experiments (red).

6.5 Randomly generated TNF-α stimulation protocols

In order to find TNF-α stimulation protocols that could potentially increase the number of identifiable parameters we randomly generated 1000 TNF-α stimulation patterns. We considered protocols consisting of 16 time intervals, each 10 minutes long. The TNF-α concentration in the first interval was drawn with equal probability from the allowed concentrations $1, 2, 3, 4, 5, 6, 7, 8, 9, 10$ ng/ml. For every remaining interval we drawn 0 ng/ml with probability 0.6 and each of the concentrations $1, 2, 3, 4, 5, 6, 7, 8, 9, 10$ with 0.04 probability.

We found that adding new protocols cannot increase the number of identifiable parameters. In Figure 4 we show the distribution of the number of identifiable parameters for the literature experiments considered jointly with one randomly generated experiment. The number of identifiable parameter does not increase. It is the result of some parameters being highly correlated with the remaining model parameters in the randomly generated experiments. Histograms show CCs between each of the selected 19 highly correlated parameters and the remaining parameters in the sampled 1000 random experiments.

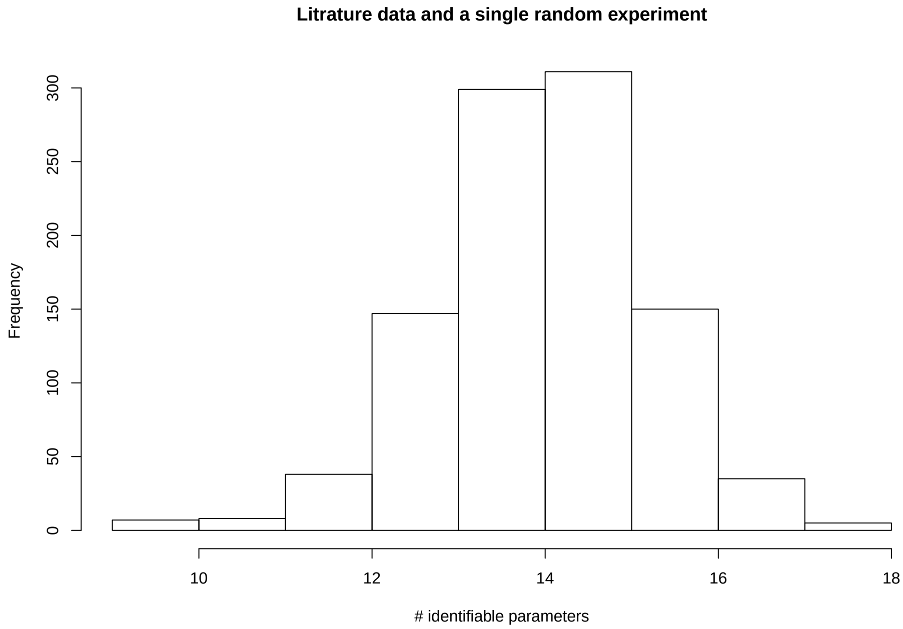

Figure 4: Histogram of the number of (δ, ζ)-identifiable parameters for the NF-κB model using literature data and a randomly generated TNF-α stimulation protocols. Thresholds $\delta = 0.95$ and $\zeta = 1$ were used.

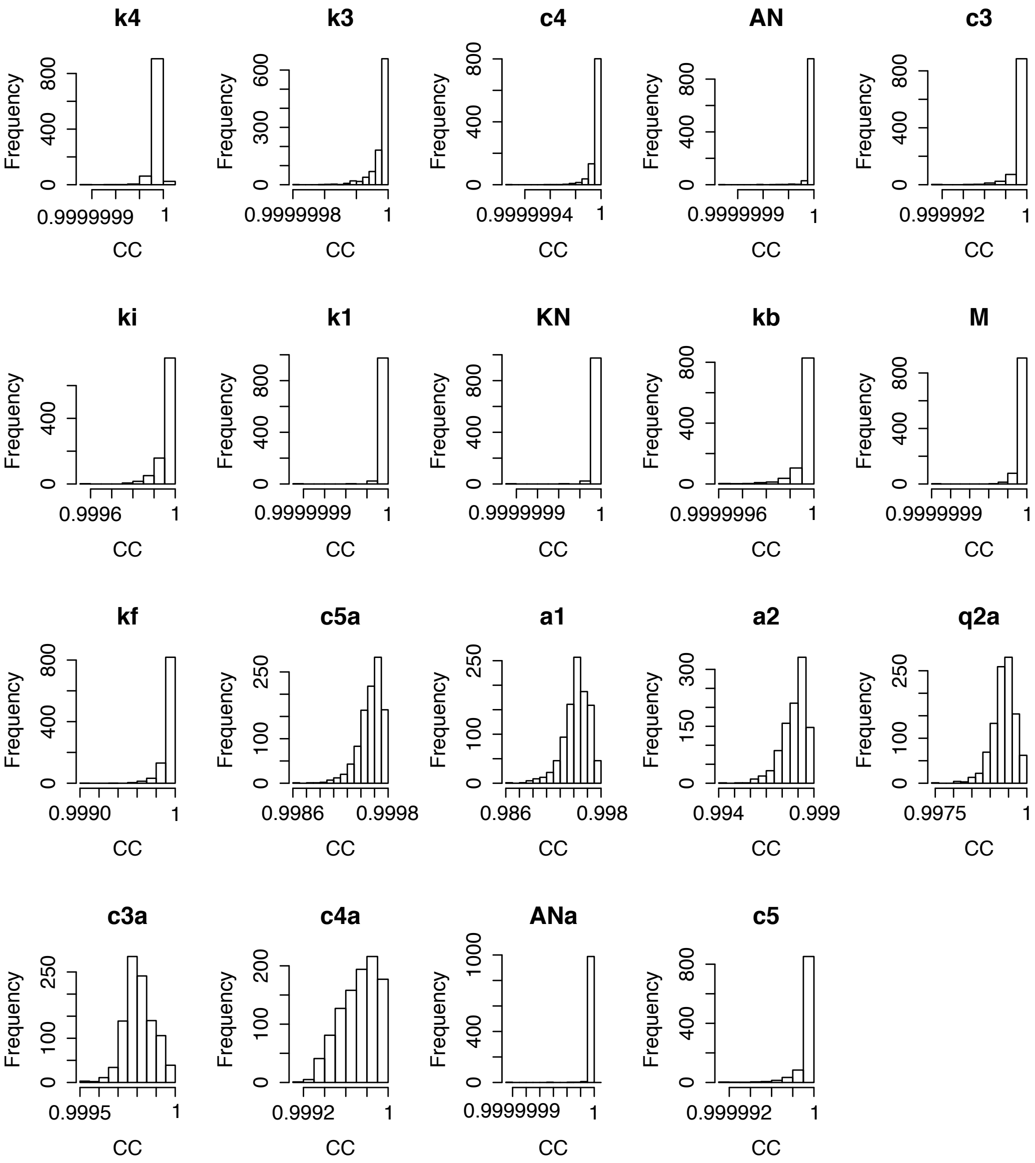

Figure 5: Histograms of correlations between the indicated parameter and remaining model parameters of the NF-κB model in the 1000 randomly generated TNF-α stimulation protocols.

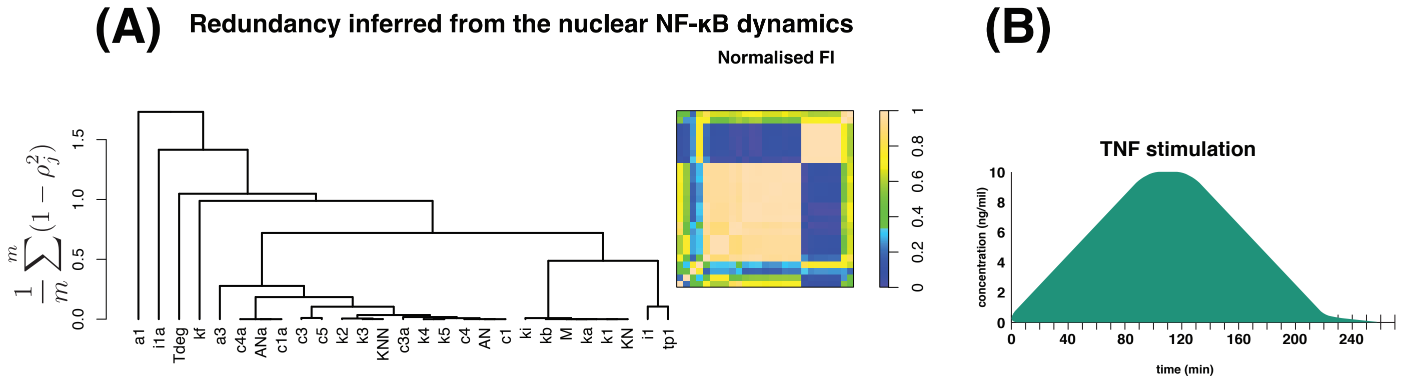

Figure 6: This Figure corresponds to Figure 3 in MP. **(A)** Dendrogram of parameters similarity in the NF-κB system calculated under the assumption that the nuclear concentration of the NF-κB is the only observable. **(B)** The TNF-α stimulation profile used in calculation of (A) and of Figure 4 in MP.

Parameter	Description
Cell parameters	
KN=10^5	total number of IKKK kinase molecules
KNN=2*10^5	total number of IKK kinase molecules
kv=5	cytoplasm to nuclear volume ratio
M=2000	number of TNF receptors
AN=2	number of A20 alleles
ANa=2	number of IKBa alleles
TNFα receptor kinetics	
kb=1.2*10^-5 s^{-1} $(ng/ml)^{-1}$	receptor activation rate
kf=1.2*10^-3 s^{-1}	receptor inactivation rate
TNFα receptor activation and signal transduction pathway	
ka=10^-5 s^{-1}	IKKK kinase activation rate
ki=0.01 s^{-1}	IKKK kinase inactivation rate
k1=6*10^-10 s^{-1}	IKKn activation caused by active IKKK
k2=10000 s^{-1}	IKKa inactivation caused by A20
k3=0.002 s^{-1}	IKKa inactivation
k4=0.001 s^{-1}	IKKi ->IKKii transformation

Parameter	Description
q2=10^-6 s^{-1}	inducible detaching from A20 site
q2a=10^-6 s^{-1}	IkBa inducible detaching from IkBa site
Tdeg=2*10^-4 s^{-1}	TNF loss
A20 and IκBα synthesis	
a1=5*10^-7 s^{-1}	IkBA NFkB association
a2=10^-7 s^{-1}	IkBa phosphoryation due to action of IKKa
a3=5*10^-7 s^{-1}	(IkBa|NFkb) phosphorylation due to action of IKKa
c1=0.1 s^{-1}	inducible A20 mRNA synthesis
c1a=0.1 s^{-1}	inducible IkBa mRNA synthesis
c3=0.00075 s^{-1}	A20 mRNA degradation rate
c3a=0.00075 s^{-1}	IkBa mRNA degradation rate
c4=0.5 s^{-1}	A20 translation rate
c4a=0.5 s^{-1}	IkBa translation rate
c5=0.0005 s^{-1}	A20 degradation rate
IκBα interactions	
c5a=0.0001 s^{-1}	IkBa degradation rate
c6a=0.00002 s^{-1}	spontaneous (IkBa|NFkB) degradation of IkBa complexed to NF-kB
tp=0.01 s^{-1}	degradation of free phospho-IkBa
tp1=0.01 s^{-1}	degradation of phospho-IkBa complexed to NFkB
Nuclear shuttling	

Parameter	Description
k5=0.001 s^{-1}	IKKi->IKKn transformation
ka20=10^5 s^{-1}	A20 TNF receptor block
q1=4*10^-7 s^{-1}	NF-kB ataching at A20 site
q1a=4*10^-7 s^{-1}	NF-kB ataching at IkBa site

Parameter	Description
e1a=0.005 s^{-1}	%default 0.005 - IkBa nuclear export
e2a=0.05 s^{-1}	(IkBa|NFkB) nuclear export
i1=0.01 s^{-1}	NFkB nuclear import
i1a=0.002 s^{-1}	IkBa nuclear import

Table 1: Parameters used to analyse the NF- κB system. The following initial conditions were used: $y_{14}(0) = 10^5$ (NF- κB cytoplasmic complex); $y_2(0) = 2 \cdot 10^5$ (initial IKKn, total IKK is kept constant) ; $y_{11}(0) = 0.14 \cdot y0(14)$ (free cytoplasmic IκBα protein); $y_{12}(0) = 0.06 \cdot y0(14)$ (free nuclear IκBα protein); $y_{13}(0) = 10$ (I κBα mRNA); $y_{10}(0) = 10$ (A20 mRNA); $y_9(0) = 10000$ (A20 protein); $y_8(0) = 1$ (free nuclear NF- κB). The parameter values and initial conditions have been taken directly from [16].

Protocol	Strain	Measured variables	Model variables	Times (min)	Figures	Source
constant TNF 20 ng/ml	WT	IKKA	y(3)	0,1,2,5,10,15,20,30,60	1A	Delhase 1999
constant TNF 10 ng/ml	TW and A20 -/-	NF-kB nuclear	y(8)	0,10,20,30,60,90,120, 180	1A	Lee 2000
		IkBa mRNA	y(13)	0,30,60,90,120,150	1C	Lee 2000
		IkBa protein	y(5)+y(6)+y(11) +y(12)+y(14) +y(15)	0,15,30,60,90	1B	Lee 2000
		IkBa protein - P	y(5)+y(6)	0,15,30,60,90	1D	Lee 2000
		IKK A	y(3)	0,10,30,60,90,120	1E	Lee 2000
constant TNF 10 ng/ml	WT	NF-kB nuclear<br>IKBa protein	y(8)<br>y(5)+y(6)+y(11) +y(12)+y(14) +y(15)	0,2,5,10,15,30,45,60,7 5,90,105,120,150,180, 210,240,270,300,330,3 60	2E	Hoffman 2002
single TNF 10ng/ml pulses of 5, 15, 30, 60 min	WT	NF-kB nuclear	y(8)	0,2,5,10,15,30,45,60,7 5,90,105,120,150,180	2E	Hoffman 2002
constant TNF 10 ng/ml		NF-kB nuclear	y(8+y(15)	every 3 minutes till 10h	2C	Nelson 2004
		IkBa protein<br>IkBa protein	y(5)+y(6)<br>y(5)+y(6)+y(11) +y(12)+y(14)+y (15)	0,5,10,15,20,30,60,90, 120,150,180,210,240,2 70	4D	Nelson 2004
single TNF 1ng/ml pulses of 45 min	WT and A20 -/-	NF-kB nuclear<br>IKKA	y(8)<br>y(3)	0,5,10,15,30,45,60,90, 120,180,240	3A,3C	Werner 2005
double TNF 20ng/ml pulses of 5 min separated by 30,60,90,120, 150,180 minutes intervals	WT	IKKa	y(3)	10 minutes after pulse (for each protocol different time)	3G	LIpniacki 2007
single TNF 1ng/ml pulses of 1,2,5,15 min	WT	NF-kB nuclear<br>IKKA	y(8)<br>y(3)	0,5,10,30,60	1D	Werner 2008

Protocol	Strain	Measured variables	Model variables	Times (min)	Figures	Source
single TNF 1ng/ml pulses of 1 min	WT	IkBa mRNA	y(13)	15,30,60,120	1E	Werner 2008
constant TNF 1 ng/ml	WT and A20 -/-	A20 mRNA<br>IkBa mRNA	y(10)<br>y(13)	15, 30,45,60,90,120,180,240,360,480,600,720	3A	Werner 2008
		NF-kB nuclear	y(8)	0,15,30,45,60,90,120	3B, 3D	Werner 2008
single TNF 1ng/ ml pulses of 5, 15, 45 min	WT and A20 -/-	NF-kB nuclear	y(8)	30,60,120,180	4B	Werner 2008
		IKKa	y(3)	30,60,120,180	S4B	Werner 2008
single TNF 10 ng/ml pulses of 5, 15, 30, 60 min	WT	NF-kB nuclear	y(8)+y(15)	every 3 min for 4h	S7	Ashall 2009
constant TNF 1 ng/ml	WT	NF-kB nuclear	y(8)+y(15)	every 3 min for 6 h	1A,1C, 3C	Ashall 2009
three TNF 10 ng/ml pulses of 5 min separated by 60, 100, 200 minutes intervals	WT	NF-kB nuclear	y(8)+y(15)	every 3 min for 10 h	2A	Ashall 2009
double TNF 10ng/ml pulses of 5 min separated by 60	WT	IkBa protein<br>IkBa protein-P	y(5)+y(6)+y(11) +y(12)+y(14) +y(15)<br>y(5)+y(6)	0,5,20,60,65,80,120,160	S5A	Ashall 2009
double TNF 10ng/ml pulses of 5 min separated by 100	WT	IkBa protein<br>IkBa protein-P	y(5)+y(6)+y(11) +y(12)+y(14) +y(15)<br>y(5)+y(6)	0,5,20,60,100,105, 120, 160	S5B	Ashall 2009
double TNF 10ng/ml pulses of 5 min separated by 200	WT	IkBa protein<br>IkBa protein- P	y(5)+y(6)+y(11) +y(12)+y(14) +y(15)<br>y(5)+y(6)	0,5,20,60,200,205,220, 260	S6	Ashall 2009
constant TNF 10 ng/ml	WT	IkBa protein	y(5)+y(6)+y(11) +y(12)+y(14) +y(15)	0,15,30,60,120,240,360,480	S11	Ashall 2009

Protocol	Strain	Measured variables	Model variables	Times (min)	Figures	Source
constant TNF 10 ng/ml	WT	IkBa mRNA	y(13)	0,15,30,60,120,240,480	S12	Ashall 2009
		IkBa gene state	y(18)	20,40,60,80,120,150,180,210	S12	Ashall 2009
single TNF 10ng/ml pulses of 5 min	WT	IkBa gene state	y(18)	20,40,60,80,120,150,180,210	S1A	Ashall 2009
double TNF 10ng/ml pulses of 5 min separated by 100 minutes	WT	IkBa gene state<br>A20 mRNA<br>IkBa mRNA	y(18)<br>y(10)<br>y(13)	0,20,40,60,100,120,140,160, 200	Fig.S23A, S23B	Ashall 2009
multiple TNF 10ng/ml pulses of 5 min separated by 100 minutes for 6h	WT	A20 mRNA<br>IkBa mRNA	y(10)<br>y(13)	0,15,115,215,315,415, 515, 615	S23B	Ashall 2009
multiple TNF 10ng/ml pulses of 5 min separated by 200 minutes for 6h	WT	A20 mRNA<br>IkBa mRNA	y(10)<br>y(13)	0,15,115,215,315,415, 515, 615	S23B	Ashall 2009
constant TNF 1ng/	WT	NF-kB nuclear	y(8)+y(15)	every 6 minutes for 6h	1C, 1D	Tay 2010
		IkBa mRNA<br>A20 mRNA	y(13)<br>y(10)	0,30,60,120, 240, 6h, 8h,10,12h	2A, 2B	Tay 2010

Table 2: Summary of the experiments on the NF-κB system published in [17–25]. The columns contain: *Protocol* - TNF-α stimulation temporal profile; *Strain* - cell type i.e either wild type (WT) or A20 knockout (A20 -/-); *Measured variables* - description of experimentally measured variables; *Model variables* - the corresponding symbol of measured variables; *Times* - measurement times; *Figures* - the figure number where data are presented in the paper given in the column *Source*.

6.6 Experiments to increase the number of identifiable parameters

Here we provide technical details regarding experiments that increase the number of identifiable parameters as well as describe reasoning that lead us to propose them. Among non-identifiable parameters in Figure 4A in MP, we have selected 6 parameters ki, KN, ka, $c3$, $c4$ and $c3a$ to be identified. As these refer to different modules of the pathway we divide them into two subsets: ki, KN, ka and $c3$,$c4$, $c3a$. Experiments to estimate parameters of each subset are described separately below.

6.6.1 Experimental design for parameters ki, KN, ka

Among equations (31) parameters ki, KN and ka are only involved in the equation of phosphorylated IKKK (y_1)

$$\dot{y}_1 = ka\ y_{16}\ (KN - y_1)\ ka20/(ka20 + y_9) - ki\ y_1. \qquad (32)$$

In order to make inference for this equation independently of other equations variables y_1, y_9, y_{16} must be measured. Therefore we assume these variables are measured in WT cells for 60 minutes every 5 minutes. We also assume the parameter $k20$ is estimated along with the other three parameters. Throughout first 5 minutes of 60 minutes long experiment cells are stimulated with 10ng TNF-α. The dendrogram that corresponds to such experiment is presented in Figure 7A. The parameters exhibit high correlation as can be expected from the structure of the equation (32). In order to improve identifiability we notice that using A20 knockout cells would eliminate $k20$ from the equations. Similarly, blocking dephosphorylation with a phosphatase inhibitor would eliminate ki. Therefore measuring the assumed three variables after TNF-α stimulation in three types of cells: wild type, A20 knockout, and A20 knockout with blocked phosphatases activity, would render all parameters identifiable. This is indeed reflected in the corresponding dendrogram (Figure 7C).

Predicions are confirmed by profile likelihoods

In order to verify identifiability predictions, we generated data from the model. The generated data were used to plot profile likelihoods. There is an ideal correspondence between

what is predicted by our method and identifiability based on profile likelihoods. This is shown in Figure 7B,D.

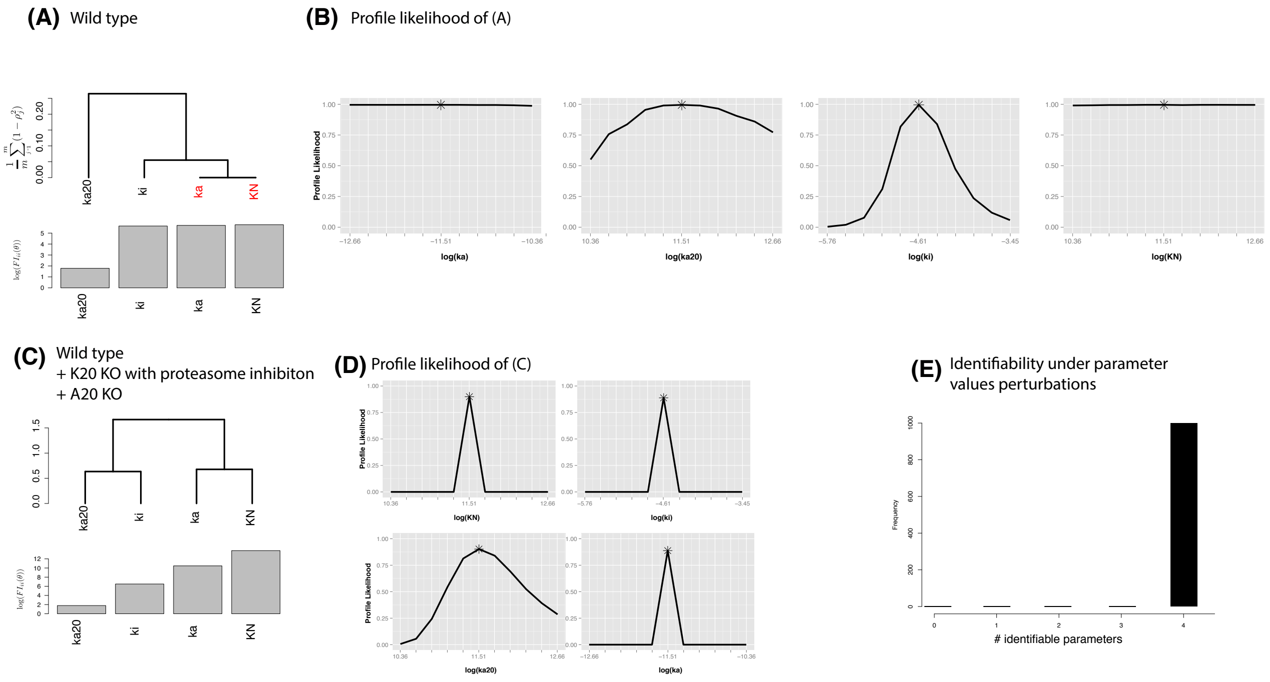

Figure 7: Dendrogram for experiments aiming to estimate parameters ki, KN, ka along with $k20$. Dynamics of IKKK activity (y_1) receptor activity (y_{16}), and cytoplasmic A20 (y_9) was assumed to be measured in (A) WT cells; (C) jointly in WT cells, A20 knockouts, and A20 knockouts with phosphatase activity inhibitor. Predictions of the dendrograms (A) and (C) are verified by profile likelihoods in (B) and (D) respectively. (E) presents histogram of the number identifiable of parameters in experiment (C) when values of the parameters $k20$, ki, KN, ka were randomly perturbed by upto 50%. This is to demonstrate that the prediction does not depend on actual value of the parameters.

Identifiability predictions do not depend on parameter values

To verify if our predictions depend on actual values of the parameters we have randomly generated 1000 sets of parameter values. For each set we calculated the number of identifiable parameters using our (δ,ζ)-criterium with $\zeta = 1$ and $\delta = 0.95$. In each of the 1000 sets all parameters were identifiable. This is depicted in the histogram (7D). Parameters were

sampled according to the formula

$$\theta_i^{(j)} = \theta_i^{(0)} \cdot 1.5^{(2*U-1)},$$

where $\theta_i^{(0)}$ is the primary value of one of the four parameters, i is indexing the four parameters, j is indexing the 1000 generated sets and U is a random variable sampled from a uniform distribution on [0,1].

6.6.2 Experimental design for parameters $c3$, $c4$, $c3a$

Estimation of the parameter $c3a$, which is the degradation rate of $I\kappa B\alpha$ transcript, present in the equation

$$\text{I}\kappa\text{B}\alpha \text{ transcript} \qquad \frac{y_{13}}{dt} = c1a\ y_{18} - c3a\ y_{13}$$

is relatively straightforward [26, 27]. We assume that after 10 min long 10ng TNF-α stimulation and 50 minutes of waiting for transcripts to be produced transcription is blocked with a transcription inhibitor. The above equation is then fitted to data assuming $c1a = 0$. In our numerical simulations we assumed transcripts were measured every 5 minutes for 2h starting at 1h after initial TNF-α stimulation.

Parameters $c3$,$c4$ denote degradation of A20 transcript, translation and degradation of A20 protein, respectively. Equations depended on these parameters are

$$\begin{aligned} \text{cytoplasmic A20} \quad \frac{y_9}{dt} &= c4\ y_{10} - c5\ y_9, \\ \text{A20 transcript} \quad \frac{y_{10}}{dt} &= c1\ y_{17} - c3\ y_{10}. \end{aligned} \tag{33}$$

We assume parameter $c5$ is estimated along with $c3$ and $c4$. In order to obtain information about translation rate and degradation rates we considered again cells being stimulated for 10 minutes with 10ng TNF-α. In order to avoid dissect the equation from the rest of the system we assume transcription is blocked just after 10 min of TNF-α stimulation. Three modified versions of this experiments were jointly required to obtain sufficiently decelerated parameters. In version one only A20 transcript is measured, in version two only A20 protein is quantified, in the third scenario both A20 mRNA and protein are measured. Such combination of measurements allowed us to break parameter correlations. This is depicted in Figure 8. Parameters of this experiment are plotted jointly with the experiment to estimate ki, KN, ka. The dendrogram plotted for literature experiments together with the proposed experiments is plotted in Figure 4B in MP.

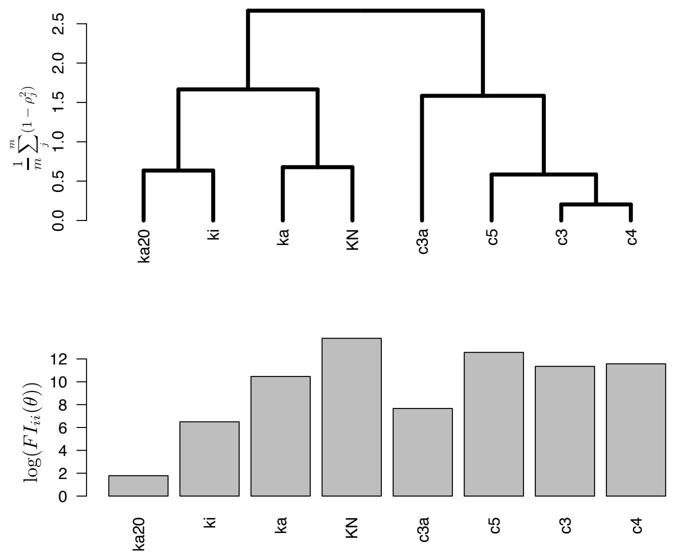

Figure 8: Dendrogram corresponding to the experiments proposed in sections 6.6.1 and 6.6.2.

7 Analysis of the MAPK signalling model

In order to demonstrate that the described methodology is suitable for models that involve hundreds of parameters, and to verify if correspondence between similarity and network topology is not limited to the NF-κB system, we analyzed a computational model of dynamics of the MAP kinase cascade activated by surface and internalized EGF receptors [28]. The EGF receptor belongs to the tyrosine kinase family of receptors and is expressed in virtually all organs of mammals. It plays a complex role during embryonic and postnatal development and in the progression of tumor. Binding of EGF to the extracellular domain of the EGF receptor initialize complex process. These lead to phosphorylation, transmission of conformational change, and proximal translocation to membrane-associated target molecules. It induces two pathways: Shc-dependent and Shc-independent, leading to activation of Ras-GTP and eventually MAP kinase cascade through the kinases Raf, MEK and ERK. Activated ERK phosphorylates and regulates several cellular proteins and nuclear transcription factors.

One of the widely accepted mathematical models of MAPK signalling was published in [28]. It involves approximately 100 ODEs and 200 parameters. Authors optimised parameters so that the model generates behaviour observed experimentally. Detailed analysis of the model is beyond the scope of this paper. The dendrogram plotted for this model (Figure 9), reveals that correspondence between network topology is not limited to the NF-κB model, but is likely to be more general feature of biochemical models. The latter statement however requires further verification. Parameters in the dendrogram 9 are colour coded and correspond to the arrows in the networks' schematics in Figure 10. Clearly clustered parameters describe functional modules proximate in the netwok's structure.

Even though the model involves 200 parameters the dendrogram can be computed within minutes on a standard desktop machine.

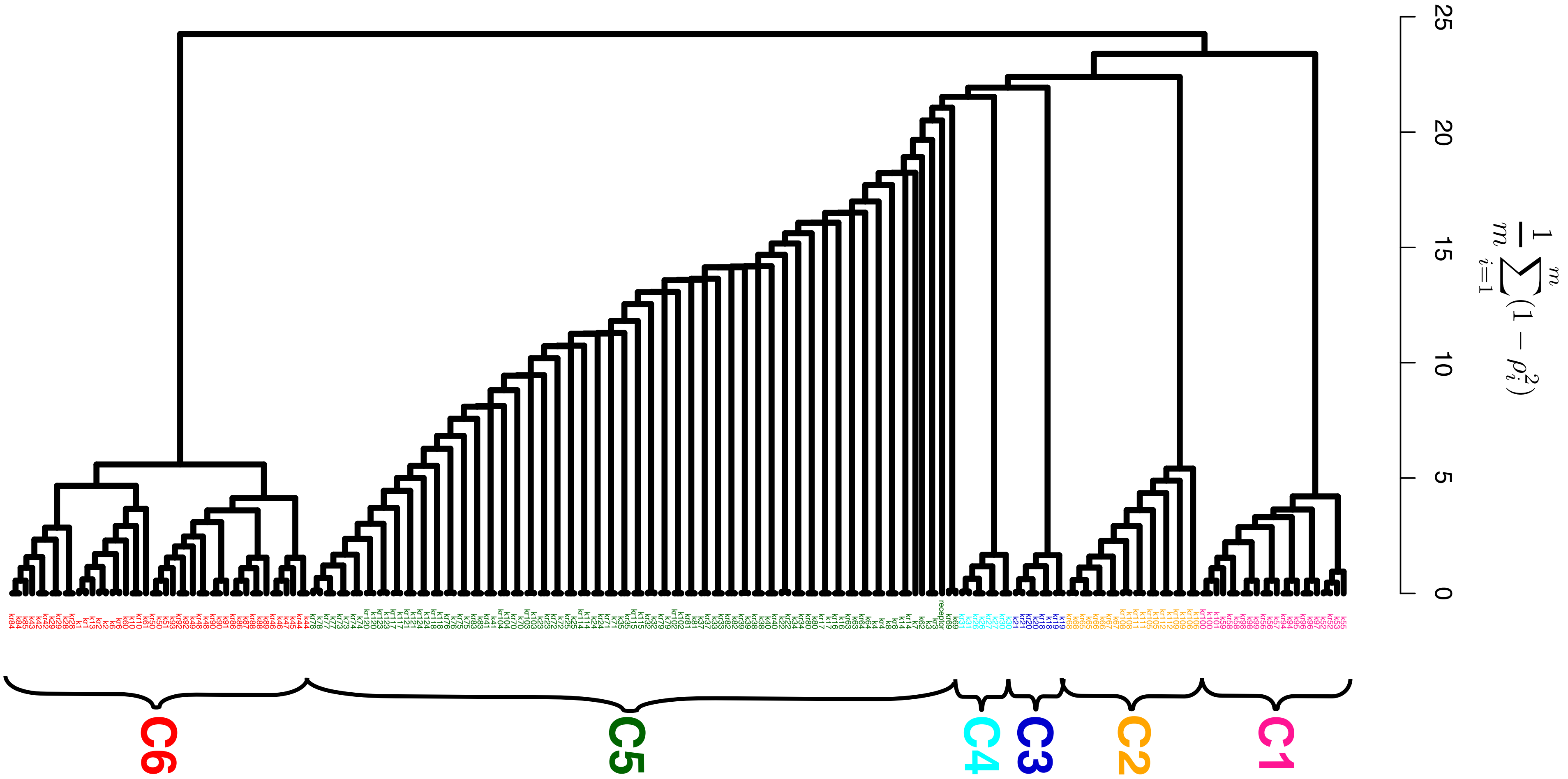

Figure 9: Dendrogram of MAPK signalling model [28]. The model was imported as an SMBL file directly from the BioModels database under the ID *B*IOMD0000000019 - Schoeberl2002 - EGF MAPK Clusters are described in the caption of Figure 10.

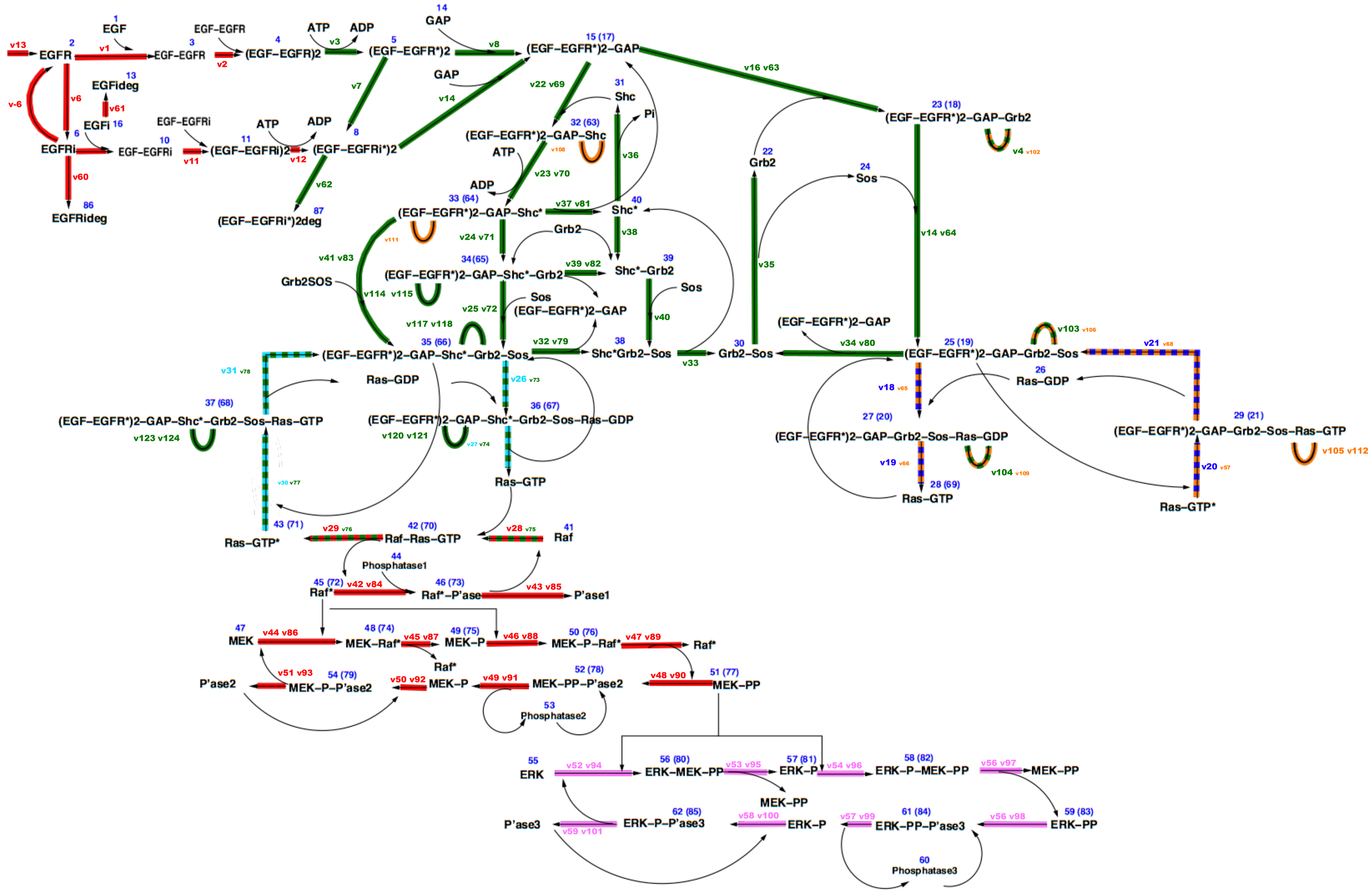

Figure 10: Structure of the MAPK signalling model [28]. Reaction arrows and labels are colour coded as in the corresponding dendrogram. The similarity of the model parameters highly resembles topology of the network. Parameter clusters correspond to the following functional modules: cluster **C1** - ERK activation; clusters **C2** and **C3** - part of Shc-dependent Ras-GTP activation pathway; cluster **C4** - final part of Shc-independent Ras-GTP activation pathway without internalization mechanism; cluster **C5** - complex (EGF-EGFR)2 phosphorylation and Shc-independent Ras-GTP activation pathway and cluster **C6** - binding EGF to EGF Receptor and MEK kinase activation pathway.

References

1. Rand, D. A., 2008. Mapping the global sensitivity of cellular network dynamics. *Journal of The Royal Society Interface* 5:S59.

2. Scharf, L. L., 1991. Statistical signal processing, volume 98. Addison-Wesley Reading.

3. Shannon, E., 1948. A mathematical theory of evidence: Bellsyt. *Techn. Journal* 27:379–423.

4. Bernardo, J., and A. Smith, 2001. Bayesian theory. *Measurement Science and Technology* 12:221–222.

5. Johnson, R. A., 1970. Asymptotic expansions associated with posterior distributions. *The annals of mathematical statistics* 41:851–864.

6. Van der Vaart, A. W., 2000. Asymptotic statistics, volume 3. Cambridge university press.

7. Kay, J., 1992. Feature discovery under contextual supervision using mutual information. *In* Neural Networks, 1992. IJCNN., International Joint Conference on. IEEE, volume 4, 79–84.

8. Girolami, M., and B. Calderhead, 2011. Riemann manifold langevin and hamiltonian monte carlo methods. *Journal of the Royal Statistical Society: Series B (Statistical Methodology)* 73:123–214.

9. Chu, Y., and J. Hahn, 2007. Parameter set selection for estimation of nonlinear dynamic systems. *AIChE journal* 53:2858–2870.

10. Chu, Y., and J. Hahn, 2008. Parameter set selection via clustering of parameters into pairwise indistinguishable groups of parameters. *Industrial & Engineering Chemistry Research* 48:6000–6009.

11. Chu, Y., and J. Hahn, 2012. Generalization of a parameter set selection procedure based on orthogonal projections and the D-optimality criterion. *AIChE Journal* 58:2085–2096.

12. Kreutz, C., and J. Timmer, 2009. Systems biology: experimental design. *FEBS Journal* 276:923–942.

13. Raue, A., C. Kreutz, T. Maiwald, J. Bachmann, M. Schilling, U. Klingmüller, and J. Timmer, 2009. Structural and practical identifiability analysis of partially observed dynamical models by exploiting the profile likelihood. *Bioinformatics* 25:1923–1929.

14. Raue, A., J. Karlsson, M. P. Saccomani, M. Jirstrand, and J. Timmer, 2014. Comparison of approaches for parameter identifiability analysis of biological systems. *Bioinformatics* btt006.

15. Venzon, D., and S. Moolgavkar, 1988. A method for computing profile-likelihood-based confidence intervals. *Applied Statistics* 87–94.

16. Murphy, S. A., and A. W. Van der Vaart, 2000. On profile likelihood. *Journal of the American Statistical Association* 95:449–465.

17. Tay, S., J. Hughey, T. Lee, T. Lipniacki, S. Quake, and M. Covert, 2010. Single-cell NF-[kgr] B dynamics reveal digital activation and analogue information processing. *Nature* 466:267–271.

18. Delhase, M., M. Hayakawa, Y. Chen, and M. Karin, 1999. Positive and negative regulation of IκB kinase activity through IKKβ subunit phosphorylation. *Science* 284:309–313.

19. Lee, E., D. Boone, S. Chai, S. Libby, M. Chien, J. Lodolce, and A. Ma, 2000. Failure to regulate TNF-induced NF-kappa B and cell death responses in A20-deficient mice. *Science Signalling* 289:2350.

20. Hoffmann, A., A. Levchenko, M. L. Scott, and D. Baltimore, 2002. The Ikappa B-NF-kappa B Signaling Module: Temporal Control and Selective Gene Activation. *Science* 298:1241–1245.

21. Nelson, D., A. Ihekwaba, M. Elliott, J. Johnson, C. Gibney, B. Foreman, G. Nelson, V. See, C. Horton, D. Spiller, et al., 2004. Oscillations in NF-{kappa} B signaling control the dynamics of gene expression. *Science Signalling* 306:704.

22. Werner, S., D. Barken, and A. Hoffmann, 2005. Stimulus specificity of gene expression programs determined by temporal control of IKK activity. *Science Signalling* 309:1857.

23. Lipniacki, T., K. Puszynski, P. Paszek, A. Brasier, and M. Kimmel, 2007. Single TNFα trimers mediating NF-κB activation: stochastic robustness of NF-κB signaling. *BMC bioinformatics* 8:376.

24. Werner, S., J. Kearns, V. Zadorozhnaya, C. Lynch, E. O'Dea, M. Boldin, A. Ma, D. Baltimore, and A. Hoffmann, 2008. Encoding NF-κB temporal control in response to TNF: distinct roles for the negative regulators IκBα and A20. *Genes & development* 22:2093–2101.

25. Ashall, L., C. Horton, D. Nelson, P. Paszek, C. Harper, K. Sillitoe, S. Ryan, D. Spiller, J. Unitt, D. Broomhead, et al., 2009. Pulsatile stimulation determines timing and specificity of NF-{kappa} B-dependent transcription. *Science Signalling* 324:242.

26. Finkenstadt, B., E. Heron, M. Komorowski, K. Edwards, S. Tang, C. Harper, J. Davis, M. White, A. Millar, and D. Rand, 2008. Reconstruction of transcriptional dynamics from gene reporter data using differential equations. *Bioinformatics* 24:2901.

27. Finkenstädt, B., D. J. Woodcock, M. Komorowski, C. V. Harper, J. R. Davis, M. R. White, D. A. Rand, et al., 2013. Quantifying intrinsic and extrinsic noise in gene transcription using the linear noise approximation: An application to single cell data. *The Annals of Applied Statistics* 7:1960–1982.

28. Schoeberl, B., C. Eichler-Jonsson, E. D. Gilles, and G. Müller, 2002. Computational modeling of the dynamics of the MAP kinase cascade activated by surface and internalized EGF receptors. *Nature biotechnology* 20:370–375.
