# Supplementary material for: Clustering reveals limits of parameter identifiability in multi-parameter models of biochemical dynamics
Source: BMC Syst Biol. 2015 Sep 29;9:65. doi: 10.1186/s12918-015-0205-8 (PMC4587803; doi:10.1186/s12918-015-0205-8)
Supplement: Additional file 2 — Open source R implementation of the clustering algorithm is available at http://sysbiosig.org/start/resources/ . (PDF 2.88 kb) [file 12918_2015_205_MOESM2_ESM.pdf]

Open source R implementation of the clustering algorithm is available at http://sysbiosig.org/start/resources/.
